# Drosophila Wnt and STAT Define Apoptosis-Resistant Epithelial Cells for Tissue Regeneration after Irradiation

**DOI:** 10.1371/journal.pbio.1002536

**Published:** 2016-09-01

**Authors:** Shilpi Verghese, Tin Tin Su

**Affiliations:** Department of Molecular, Cellular and Developmental Biology, University of Colorado, Boulder, Colorado, United States of America; Zentrum für Molekulare Biologie der Universität Heidelberg, GERMANY

## Abstract

*Drosophila melanogaster* larvae irradiated with doses of ionizing radiation (IR) that kill about half of the cells in larval imaginal discs still develop into viable adults. How surviving cells compensate for IR-induced cell death to produce organs of normal size and appearance remains an active area of investigation. We have identified a subpopulation of cells within the continuous epithelium of *Drosophila* larval wing discs that shows intrinsic resistance to IR- and drug-induced apoptosis. These cells reside in domains of high Wingless (Wg, Drosophila Wnt-1) and STAT92E (sole *Drosophila* signal transducer and activator of transcription [STAT] homolog) activity and would normally form the hinge in the adult fly. Resistance to IR-induced apoptosis requires STAT and Wg and is mediated by transcriptional repression of the pro-apoptotic gene *reaper*. Lineage tracing experiments show that, following irradiation, apoptosis-resistant cells lose their identity and translocate to areas of the wing disc that suffered abundant cell death. Our findings provide a new paradigm for regeneration in which it is unnecessary to invoke special damage-resistant cell types such as stem cells. Instead, differences in gene expression within a population of genetically identical epithelial cells can create a subpopulation with greater resistance, which, following damage, survive, alter their fate, and help regenerate the tissue.

## Abstract 2

After widespread radiation damage in the developing fruit fly, organs are formed by regeneration from an apoptosis-resistant subpopulation of cells marked by high levels of Wingless and STAT.

## Author Summary

Like other insects, *Drosophila* larvae have epithelial structures called imaginal discs that will give rise to most of the external adult structures, such as wings, limbs, or antennae; these organ precursors are formed by a single layer of epithelial cells that folds into a sac. Imaginal discs manage to regenerate efficiently if they are damaged. Previous studies have shown that dying cells produce signals that activate cell proliferation of some of their neighbors, allowing them to regenerate the disc and thereby enabling the flies to develop into normal adults. But a dedicated stem cell population that contributes to regeneration, if any, remained to be identified. Here, we report the identification of a subpopulation of cells in wing imaginal discs that is more resistant to the cytotoxic effects of radiation and drugs. We show that the protection of these cells depends on two signaling pathways—Wingless and STAT—that are conserved in humans. Following tissue damage by radiation, we observe that protected cells change their location and their identity, allowing them to fill in for dead cells in other parts of the same organ precursor. In sum, this work identified ways in which a subset of cells in *Drosophila* imaginal wing discs is preserved through radiation exposure so that they could participate in regeneration of the organ after radiation damage. We also discuss how this situation may resemble human cancers.

## Introduction

The ability to regenerate is critical for tissue homeostasis in adult organisms. Many tissues such as the gut and the skin suffer constant environmental insults that result in cell death, requiring tissue-specific stem cells to proliferate and compensate for cell loss. Two key characteristics of stem cells, namely, greater resistance to killing compared to other cells and the ability to contribute to regeneration, are also ascribed to cancer stem cells or cancer initiating cells. Specifically, tumors and blood cancers are hypothesized to contain a small population of cells with greater ability to initiate new tumors than the rest. Such cancer-initiating cells would help replenish the tumor, after therapy for example, or to produce new tumors at distant sites as metastases. Cancer-initiating cells have been identified for different types of cancers, typically by virtue of differential cell surface markers that correlate with the ability of cells with such markers to initiate cancers or tumors in animal models (for example [1,2]). Where tested, cancer-initiating cells show greater resistance to killing by cancer therapy agents, such as radiation and chemotherapy [3,4]. Understanding molecular mechanisms that underlie the increased resistance of cancer-initiating cells and their ability to contribute to regrowth is essential for optimizing anticancer treatments. In this regard, genetically tractable model systems in which cells show resistance to death induced by cytotoxic agents and contribute to regeneration would be valuable tools.

Imaginal discs in *Drosophila* larvae are precursors of adult organs. Larval imaginal discs are composed of a layer of columnar epithelial cells covered by a layer of peripodial squamous cells. Imaginal discs lack a dedicated stem cell population yet regenerate efficiently. For example, surgical ablation of up to a quarter of a leg disc still allows complete regeneration [5]. Likewise, irradiation of larvae with X-ray doses that kill up to 50% of the cells is still compatible with the production of a viable adult [6]. In studies in wing and eye imaginal discs, dying cells are found to produce mitogenic signals that result in non-autonomous activation of cell proliferation locally, up to 5 cell diameters away from the dying cells. These signals operate through JNK, Wg, and Dpp in the wing disc and Hh and EGFR in the eye disc (reviewed in [7]). Increased proliferation of nearby cells could help replace cells lost to cell death and, therefore, help regenerate the disc.

In studies of mitogenic signals that emanate from dying cells, cell death was often confined to marked clones in order to detect effects on the neighbors. In other studies in which death-inducing stimuli was applied globally, by irradiating the whole larva, for example, the resulting cell death was not homogenous across the disc. In other words, cells within the continuous single-layer epithelium of imaginal discs show unequal propensity to die (for example, see [8,9]). What makes some cells die while their neighbors survive in the face of identical external insult remains an open question, which may be directly relevant to, for example, radiation treatment of tumors.

We report here the identification of a subpopulation of cells in *Drosophila melanogaster* larval wing disc, which, we found, shows consistent resistance to ionizing radiation (IR)- and drug-induced apoptosis. These cells reside in the region of the wing disc that normally differentiates into the hinge region of the adult wing. We found that these cells are protected from IR-induced apoptosis by Wg and signal transducer and activator of transcription (STAT) and participate in tissue regeneration after radiation damage. These results illustrate that it is unnecessary to invoke special damage-resistant cell types such as stem cells for regeneration. Instead, differences in gene expression within a population of epithelial cells can create a subpopulation to fulfill this role. We discuss ways in which this situation may be analogous to human cancers.

## Results

### Cells of the Dorsal Hinge Region Are Resistant to Radiation and Drug-Induced Apoptosis

**Fig 1:**
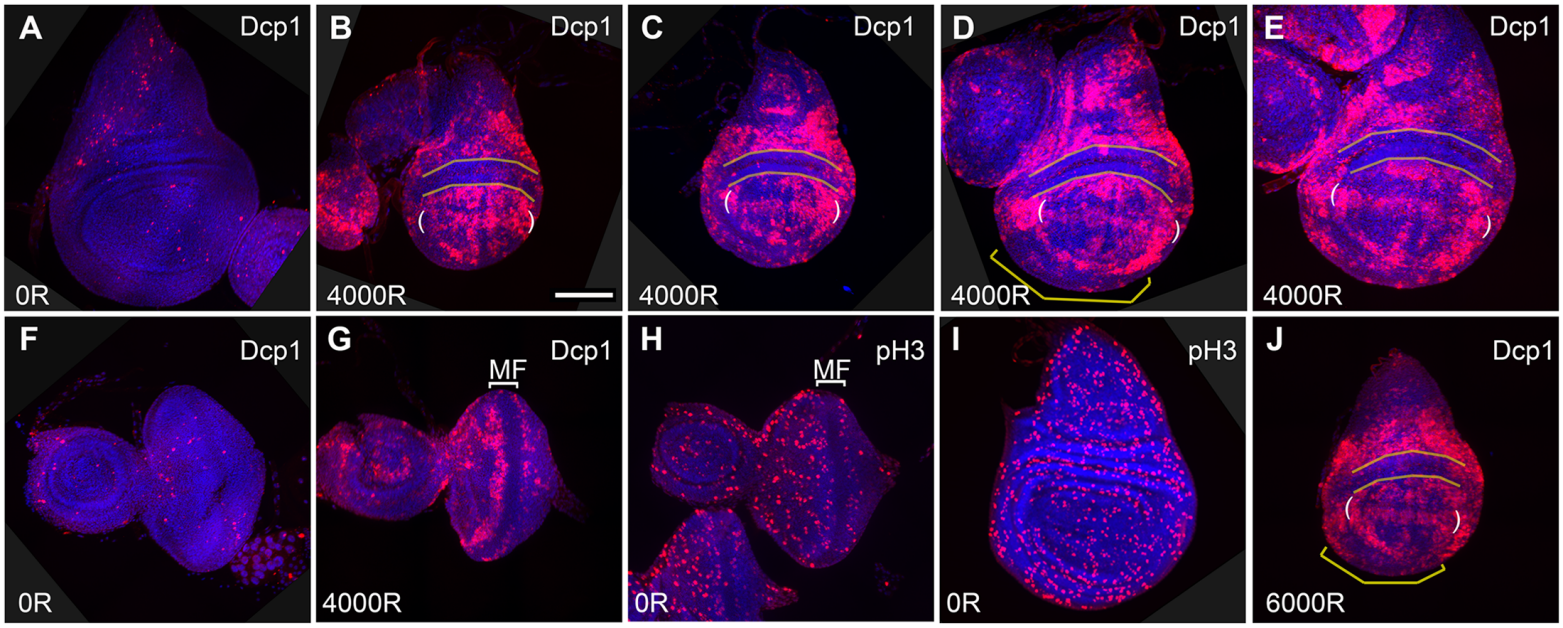
Resistance to ionization radiation-induced apoptosis varies within a larval imaginal disc. Wing (A–E, I, J) and eye-antennae (F–H) discs were dissected from 92–100 hours (h) old feeding third instar larvae 4 h after exposure to 0, 4,000, or 6,000R of X-rays, as indicated on each panel. The discs were fixed and stained with an antibody to cleaved caspase Dcp1 (A–G, J, red) or to phospho-S10-Histone H3, a mitotic marker (H and I, red). The discs were also stained for DNA (blue). Wing discs are shown with anterior left and dorsal up. Eye discs are shown with anterior left. Similar results were seen in larvae of *y*^*1*^*w*^*1118*^ and *w*^*1118*^ genotypes. Shown are tissues from *y*^*1*^*w*^*1118*^ in I and *w*^*1118*^ in all others. MF = morphogenetic furrow. Scale bar = 60 μm (I) or 45 μm (others).

It is evident in many previous studies that there are domains within the wing imaginal disc with different propensity to undergo IR-induced apoptosis (for example, [8,9]). In our experiments, apoptosis was detected by immunostaining for cleaved caspases Drice or Dcp1, or by TUNEL assay (Fig 1 and S1 Fig), in wing discs from feeding third instar larvae. For example, IR-induced apoptosis was reproducibly robust along the dorsal/ventral boundary of the wing pouch (between white brackets in Figs 1B–1E, 1J and S1). IR-induced apoptosis was reproducibly low in the dorsal part of the future wing hinge (between yellow lines in Fig 1B–1E, 1J and S1). This pattern of differential IR sensitivity was seen in wing discs of different sizes present among larvae of similar chronological age (Fig 1B–1E) and also for the entire range of X-ray doses tested, 2,000–8,000R; 4,000 and 6,000R samples are shown here. IR-resistance in this region spanned through the thickness of the disc, as seen in z-sections (S1D and S1E Fig). IR-resistance was also seen in the ventral-anterior cells of the hinge (yellow brackets in Fig 1D and 1J) but was less pronounced and less consistent than in the dorsal hinge.

IR resistance in a stripe of cells was also evident in the eye disc (bracket in Fig 1G). In this tissue, resistant cells were found within the morphogenetic furrow, a domain of cells with little or no mitotic activity (Fig 1H) and arrested in G1 [10]. This is in agreement with published studies in many systems that show an inverse correlation between mitotic proliferation and DNA damage-induced cell death [11,12]. While cell cycle phasing can explain the stripe of IR-resistant cells in the eye disc, it cannot explain the IR-resistance of future-hinge cells in the wing disc because proliferation occurs throughout the whole disc at these stages in development (Figs 1I and S1G). Therefore, we sought other explanations. We focused our analysis on the dorsal hinge region because it showed consistent resistance to IR-induced apoptosis (Fig 1B–1E and 1J), and by different detection methods (cleaved Dcp1 in Fig 1, cleaved Drice in S1A Fig, and TUNEL in S1B Fig). Because it resembles an inverted “smile,” we refer to this region as the “frown” hereafter.

It is possible that enhanced DNA repair is the reason the frown is resistant to IR-induced apoptosis. We do not favor this possibility because the same region was also resistant to a chemical microtubule depolymerizing agent maytansinol (S1C Fig), which induces robust apoptosis when fed to larvae and adults [13,14]. The resistance of frown cells to insults that act by different mechanisms, DNA damage and microtubule de-polymerization, argues for a general anti-apoptotic mechanism. This idea is also supported by the published data that heat-shock-induction of p53, in the absence of any other insult, was able to induce apoptosis throughout the wing disc except in the frown (Fig 2E in [15]). We conclude that cells of the frown are generally resistant to apoptosis.

We note a prior study of spatial differences in IR-induced apoptosis in wing discs [16]. This study focused on the wing pouch in wandering stage larvae, where cell proliferation had decreased and apoptosis was confined to inter-vein regions. Studies here are at an earlier stage in development, during which cells are still actively proliferating.

### The Frown Includes Cells with High STAT92E Activity and Is Flanked by Wg Producing Cells

**Fig 2:**
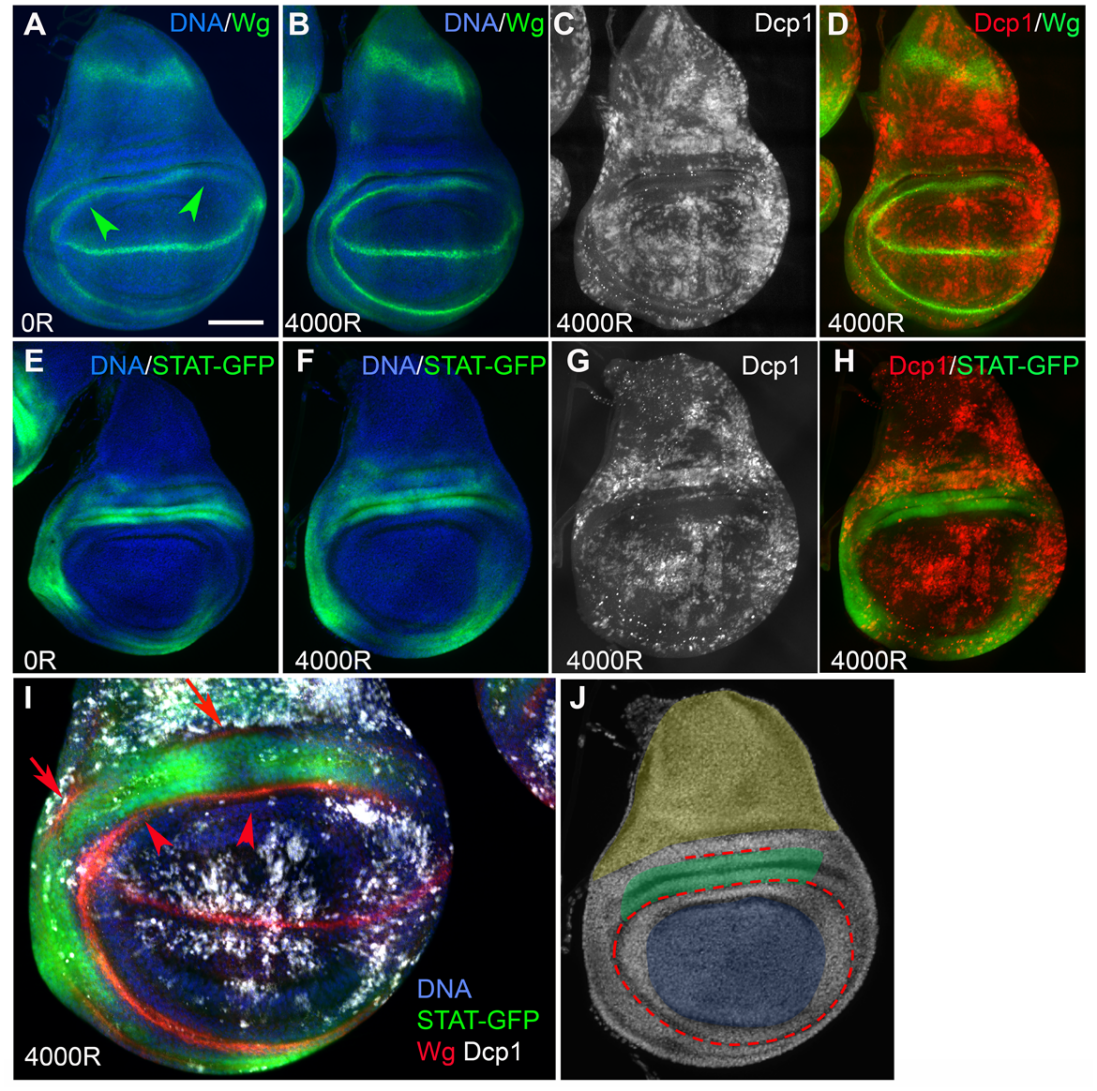
The frown includes cells with high Wg and STAT92E activity. Wing discs were dissected from 92–100 h old feeding third instar larvae 4 h after exposure to 0 or 4,000R of X-rays as indicated on each panel. The discs were fixed and stained with antibodies to Wg or cleaved Dcp1 and for DNA. Images are shown with anterior left and dorsal up. (A–D) Wing discs from wild type (*w*^*1118*^) larvae. The same disc is shown in B–D. Wg inner ring is indicated by arrowheads. (E–H) Wing disc larvae homozygous for the STAT-GFP reporter. (I) One optical slice of an irradiated disc stained to visualize all proteins of interest. Wg-IR and Wg-OR are indicated with arrowheads and arrows, respectively. (J) A wing disc imaged for DNA is shown with artificial color to indicate the regions of interest. Blue = the pouch; yellow = the notum; red = Wg-IR and Wg-OR. The frown (green) occupies two folds of cells directly dorsal to the pouch, as marked by Wg-IR. Scale bar = 25 μm in I and 50 μm in all other panels.

By examining the literature on gene expression in the wing disc, we identified Wg (Drosophila Wnt-1) and STAT92E (the sole STAT gene in *Drosophila*, “STAT” hereafter) as genes whose expression correlates spatially with the frown. Antibody staining showed that Wg-expressing cells of the so-called inner ring (Wg-IR, arrowheads in Fig 2A and 2I) lined the ventral (lower) edge of the frown (Fig 2C and 2D). The outer ring of Wg expression was less apparent at these stages but abutted the dorsal (upper) edge of the frown (Wg-OR, arrows in Fig 2I). Strikingly, the frown overlapped almost entirely with a domain of high STAT activity (Fig 2E–2H) as seen by a published GFP reporter under the control of 10 STAT binding sites [17]. This domain is comprised of two folds of epithelial cells immediately dorsal (up) to Wg-IR [18]. Thus, the frown includes cells with high STAT activity and borders on cells with high Wg expression. The area of the ventral-anterior hinge that showed increased but variable resistance to IR-induced apoptosis (yellow bracket in Fig 1D) also included cells with high STAT activity (Fig 2E and 2F), but the overlap is not as good as in the frown.

Because of the proximity between cells with high Wg and cells with high STAT-GFP, we investigated whether each influences the expression of the other. During wing development, the formation of the dorsal hinge region with high STAT activity requires Wg [18–20]. In turn, STAT is required for the growth of this region that results in the spatial separation of Wg-IR and Wg-OR [18,20]. Besides these indirect interactions, Wg and STAT pathways are thought to function in parallel and not intersect in the wing disc (reviewed in [21]). We confirmed these finding using conditional inhibition of each pathway (S2 Fig), in agreement with the published results.

### IR-Resistance in the Frown Requires Wg and STAT

**Fig 3:**
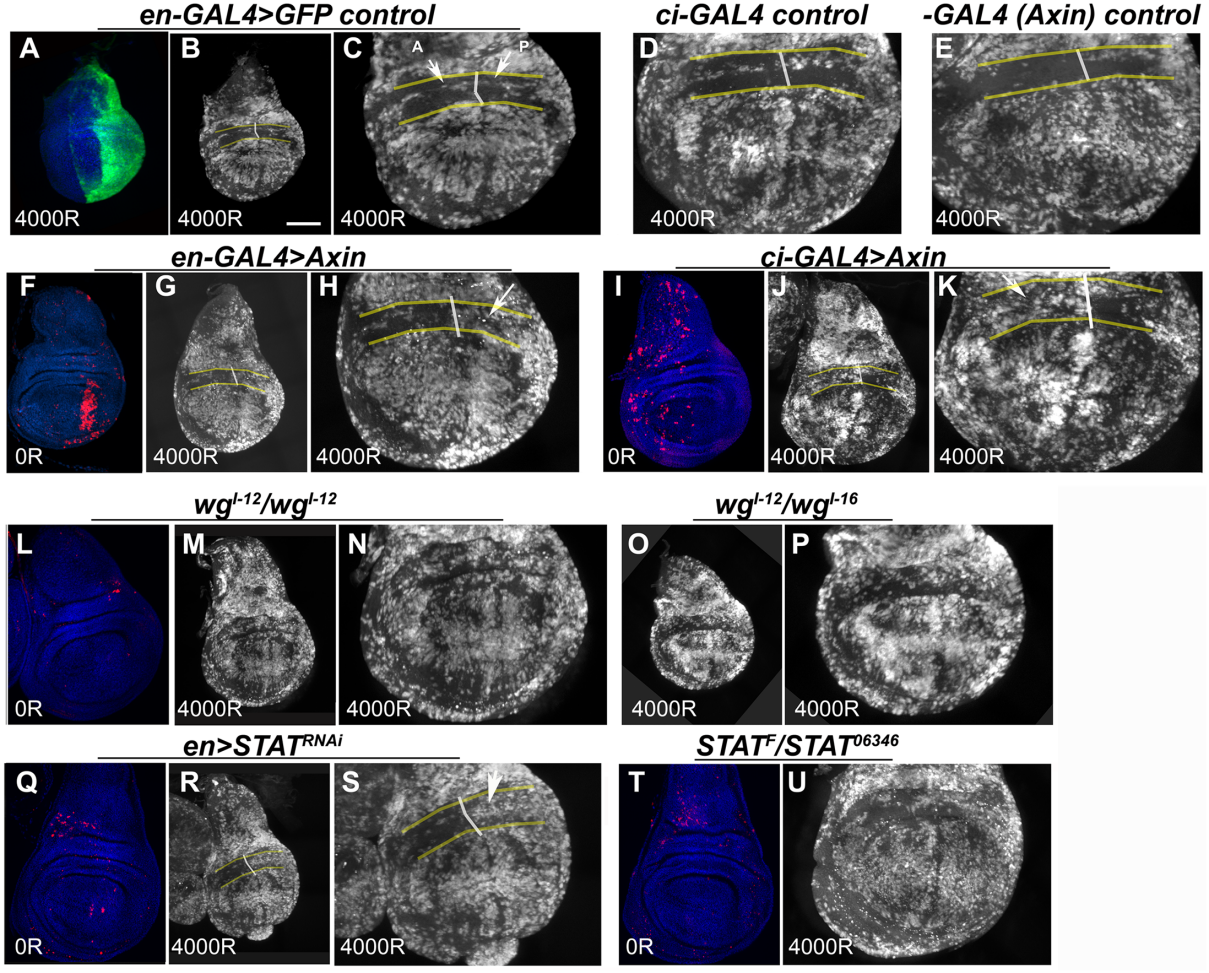
IR-resistance in the *frown* requires Wg and STAT92E. Embryos were collected at 18°C for 24 h. For inhibition of Wg (A–P), embryos were reared at 18°C for 7 d from the end of collection and shifted to 29°C for 24 h before irradiation. For STAT^RNAi^ (Q–S), embryos were reared at 18°C for 6 d and shifted to 29°C for 48 h before irradiation. For STAT^ts^ (T-U), embryos were reared at 18°C for 6 d and shifted to 29°C for 72 h before irradiation. Wing discs were dissected 4 h after exposure to 0 or 4,000R of X-rays, fixed and stained with an antibody to cleaved Dcp1 (red in F and I and white in others) and for DNA (blue). Genotypes are indicated on the panels. In addition, all larvae in GAL4 experiments carried one copy of GAL80^ts^. All transgenes were heterozygous (or hemizygous for those on X). The white lines show the A/P boundary marked by UAS-GFP or UAS-Axin-GFP expression in some discs. DNA stain was used to locate the frown (between yellow lines) as two folds of cells above the pouch (see Fig 2I and 2J). (A) An example of the P compartment marked by GFP. (B–E) Control discs showing similarly low levels of IR-induced apoptosis in A and P halves of the frown. (F–H) Expression of Axin in the P compartment caused apoptosis in the pouch on its own (F) and IR-induced apoptosis in the frown after irradiation (G, magnified in H). (I-K) Expression of Axin in the A compartment caused scattered apoptosis on its own (I) and IR-induced apoptosis in the frown after irradiation (J, magnified in K). (L–P) *wg* mutations did not cause apoptosis on their own (L) but allowed IR-induced apoptosis in the frown. M is magnified in N and O is magnified in P. (Q–U) Depletion of STAT92E did not cause apoptosis on its own (Q,T) but allowed IR-induced apoptosis in the frown. (R is magnified in S). Scale bar = 50 μm for whole discs, 25 μm for half discs, except for (U), where it is 36 μm.

We next investigated whether Wg or STAT has a role in the IR-resistance of the frown. Because of the known requirement for Wg and STAT in development, we took care to allow wing discs to develop before inhibiting each pathway conditionally (see Fig 3 legend and Materials and Methods). We expressed Axin, an inhibitor of Wg signaling (S2A Fig), in the P compartment (using *en*-GAL4) or in the A compartment (using *ci-GAL4*), in conjunction with GAL80^ts^ for conditional expression. In control discs, A and P halves of the frown showed similar, low levels of IR-induced apoptosis (Fig 3A–3E). Induction of Axin for 24 h induced scattered apoptosis but not in the frown (Fig 3F and 3I). After irradiation, apoptotic cells were observed in the half of the frown that expressed Axin but not in contralateral half (Fig 3G, 3H, 3J and 3K). These results were confirmed using homozygotes of temperature-sensitive *wg*^*1-12*^ mutants [22], which were incubated at 29°C for 24 h to inactivate Wg before irradiation. *wg*^*I-12*^ discs showed no apoptosis without IR (Fig 3L) but showed robust IR-induced apoptosis throughout the disc including the frown (Fig 3M and 3N). Trans-heterozygote of *wg*^*1-12*^ with *wg*^*I-16*^, an X-ray-induced hypomorphic allele, showed similar results, although attenuation of IR resistance was variable with this allelic combination (Fig 3O and 3P). We conclude that Wg signaling is required for the IR-resistance of frown cells. The results with Axin suggest that the requirement is cell autonomous.

We obtained similar results using TCF^DN^ to inhibit Wg signaling. As in the case of Axin, wing discs expressing *en-GAL4>UAS-*TCF^DN^ showed increased cell death in the pouch but not in the frown (S3B Fig). After irradiation, cells in the posterior half of the frown underwent apoptosis while contralateral anterior cells remained resistant (S3C and S3D Fig). These discs also showed greater intensity of DNA stain in the posterior half (S3A Fig). We do not know the reason, but this effect would be consistent with the published data that TCF^DN^ can drive G1 cells along the D/V boundary in the pouch into S phase [23]. Because the proliferation state itself could affect apoptosis, the TCF^DN^ data is harder to interpret than the data with Axin or *wg* mutants, neither of which showed altered DNA intensity (e.g., Fig 3F, 3I and 3L).

Depletion of STAT92E using a published RNAi construct (Fig 3Q–3S) or a temperature-sensitive allelic combination (Fig 3T and 3U) also increased IR-induced apoptosis in the frown, although the effect was variable with the latter treatment. We conclude that STAT92E has a role in IR resistance of the frown. To investigate the consequence of co-depleting Wg and STAT, we combined two protocols that produced the most consistent effect, STAT^RNAi^ and Axin. Under identical de-repression conditions for GAL4, incubation at 29°C for 48 h, Axin induced more apoptosis than STAT^RNAi^ in the frown (compare S4B to S4E Fig). Combining the two did not produce more apoptosis than Axin alone (compare E and K). While these results are consistent with STAT and Wg functioning in a single pathway or on the same target, we caution that analysis of genetic epistasis requires complete loss-of-function alleles. Wg and STAT are essential for wing disc development and for larval viability. As such, genetic manipulations we used that allow us to analyze wing discs in third instar larvae may not cause a complete loss of function, even under strong non-permissive conditions.

### Repression of Reaper Can Explain the Resistance to IR-Induced Apoptosis in the Frown

IR-induced apoptosis accompanies a p53-dependent increase in transcripts for pro-apoptotic genes *hid*, *reaper*, and *sickle* [24,25]. These encode SMAC/DIABLO orthologs that antagonize *Drosophila* inhibitors of apoptosis proteins 1 (DIAP1) to allow caspase activation [26]. Genetic manipulations with the potential to reduce the pool of *Drosophila* SMAC/DIABLO orthologs result in reduced and delayed induction of apoptosis after irradiation. These include heterozygosity for *hid* and chromosomal deficiencies that remove *hid* and *rpr* or *hid*, *rpr*, and *skl*. Ectopic expression of *hid* or *rpr* on its own is sufficient to induce apoptosis. These and other results led to a model in which a threshold of pro-apoptotic activity may be reached by an aggregate of Hid, Rpr, and Skl to antagonize DIAP1 and induce apoptosis [26–29].

**Fig 4:**
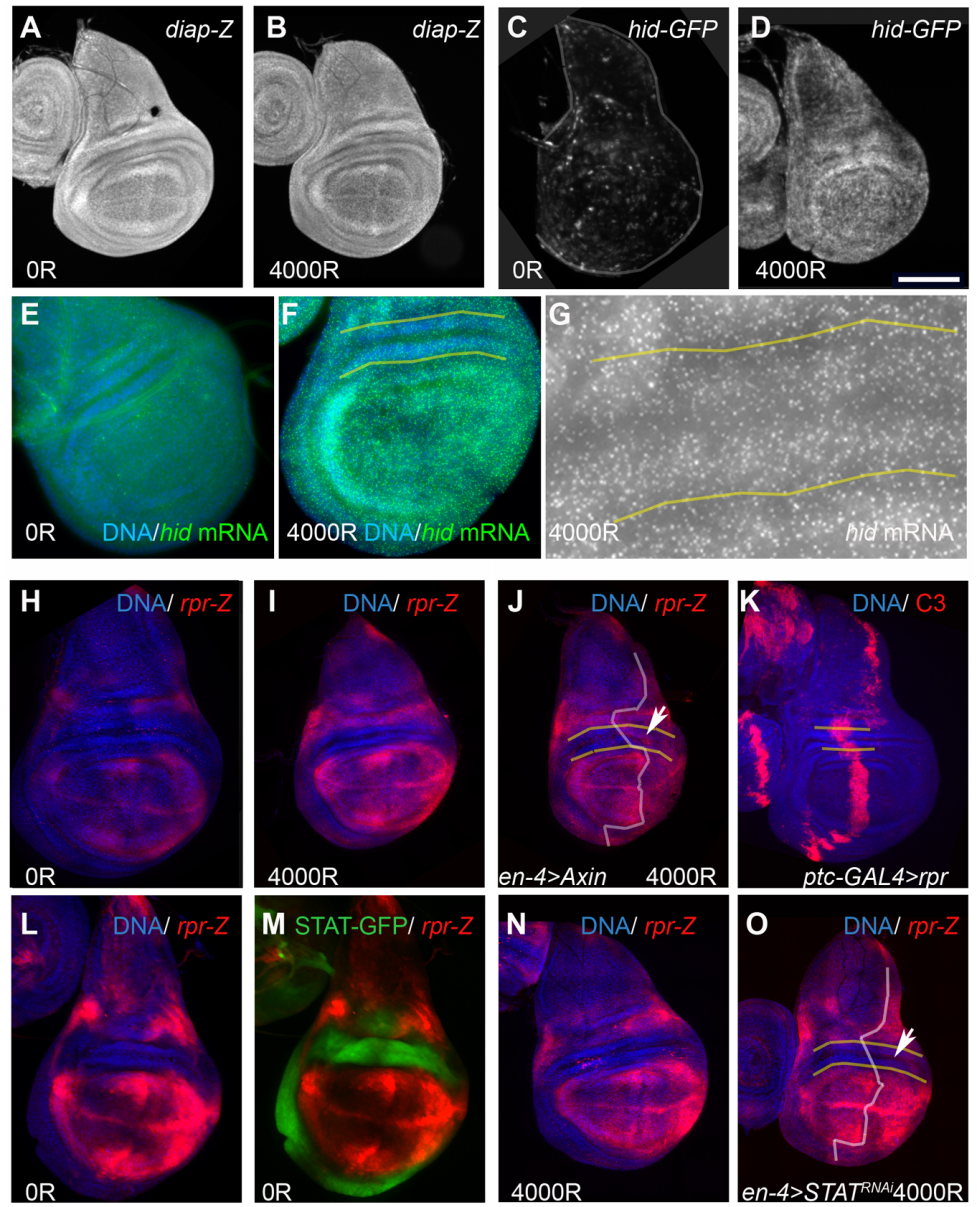
Repression of *reaper* expression in the frown. (A–I,L,M) 92–100 h old larvae were irradiated with 0 or 4,000R of X-rays. (J) Embryos were collected at 18°C for 24 h and reared at 18°C for 7 d from the end of collection and 29°C for 24 h to de-repress Axin before irradiation. (K) 92–100 h old larvae were shifted to 29°C for 12 h to de-repress UAS-*rpr* before dissection. (N,O) Embryos were collected at 18°C for 24 h and reared at 18°C for 6 d from the end of collection and 29°C for 48 h to induce STAT^RNAi^ before irradiation. Wing discs were fixed and stained with antibodies to β-galactosidase or cleaved Caspase3/Drice, imaged for GFP without staining, or processed to detect *hid* mRNA by in situ hybridization. The discs were also stained for DNA (blue). White lines marked the A/P boundary as identified by GFP expression. The frown is between yellow lines. (A,B) *diap-lacZ* transcriptional reporter 4 h after irradiation. (C,D) *hid-GFP* transcriptional reporter 4 h after irradiation. The disc in (C) is outlined. (E–G) *hid* mRNA expression by in situ hybridization in *w*^*1118*^ wing discs 2 h after irradiation. The frown and adjacent regions in (F) are shown magnified in (G) to illustrate comparable levels of *hid* both within and outside the frown. Similar results are seen at 4 h after irradiation. (H–J) *rpr-lacZ* transcriptional reporter 4 h after irradiation. (H and I) were imaged and processed identically to illustrate the induction of *rpr-lacZ* 2 h after irradiation. (K) UAS-*rpr* kills frown cells. The level of active caspase signal in the frown (between yellow lines) is well within the range seen outside the frown. (L,M) Regions of low *rpr-lacZ* reporter expression in un-irradiated discs coincide with regions of high STAT-GFP activity reporter expression. Note that the exposure for the *rpr-lacZ* reporter in (L and M) was increased compared to (H) in order to clearly discern areas of low reporter expression. (N,O) *rpr -lacZ* reporter expression in STAT^RNAi^ discs. (N) is from a control without GAL4. Scale bar = 8.3 μm (G), 25 μm (E, F), 50 μm (all other panels).

To gain insight into how cells in the frown resist IR-induced apoptosis, we monitored the expression of transcriptional reporters for DIAP1, *hid* and *rpr*. We chose DIAP1 because STAT92E has been shown to transcriptionally activate DIAP1 to resist IR-induced apoptosis in the wing pouch [30]. In these experiments, the role of STAT in the hinge was not assayed because STAT^-/-^ clones were not recovered in this region [30]. We saw no significant changes in *diap1-lacZ* reporter expression after irradiation (Fig 4A and 4B). More relevant to this study, DIAP1 protein [30] or transcriptional reporter expression (this study) were not different between the pouch and the hinge and, therefore, could not explain the differences in IR resistance. A similar result was obtained using a *hid-GFP* transcriptional reporter. This reporter is comprised of 2 kb of the *hid* 5′ regulatory sequences and could be induced by IR in a p53-dependent manner (Fig 4C and 4D; [31]). As in the case of DIAP1, we did not observe variations in *hid* reporter expression that could explain differential IR-sensitivities between the pouch and the hinge.

*rpr-lacZ* reporter expression, on the other hand, could explain the differential sensitivity of the pouch and the hinge to IR-induced apoptosis. In un-irradiated discs, *rpr-lacZ* expression was high in the pouch and parts of the notum but low in the hinge (Fig 4H, with increased gain in L). *rpr-lacZ* expression increased after irradiation as expected, but the pattern remained the same as in un-irradiated discs (Fig 4, compare H and I); that is, *rpr* expression was low in the hinge with or without IR. Induction of Axin did not alter *rpr-lacZ* expression in the hinge without IR. This is consistent with the finding that Axin did not induce apoptosis in the frown without IR (Fig 3F). After irradiation, however, we could see increased *rpr-lacZ* expression in the posterior half of the frown (arrow in Fig 4J, compared with the contralateral half of the same disc. This is consistent with the ability of Axin to promote IR-induced apoptosis in the frown (Fig 3H). Similarly, *rpr-lacZ* was also increased after irradiation in *wg*^*I-12/I-12*^ mutants (S5 Fig). Ectopic expression of *rpr* with *patched-GAL4* driven expression of a UAS transgene was sufficient to induce apoptosis in the cells of the frown to similar levels as in the pouch (Fig 4K). Collectively, these results demonstrate that cells of the frown are capable of undergoing apoptosis and suggest that they are prevented from doing so because *rpr* is repressed, at least in part by Wg signaling.

Regions of high STAT activity in the hinge correspond closely to regions of low *rpr-lacZ* expression, particularly the frown (Fig 4L–4N). Depletion of STAT by RNAi, which was sufficient to promote IR-induced apoptosis (Fig 3R and 3S), however, had little effect on *rpr-lacZ* expression (representative disc shown in Fig 4O). We conclude that while STAT acts to prevent IR-induced apoptosis in the frown (Fig 3), this effect was likely to be through another target besides *rpr*.

**Fig 5:**
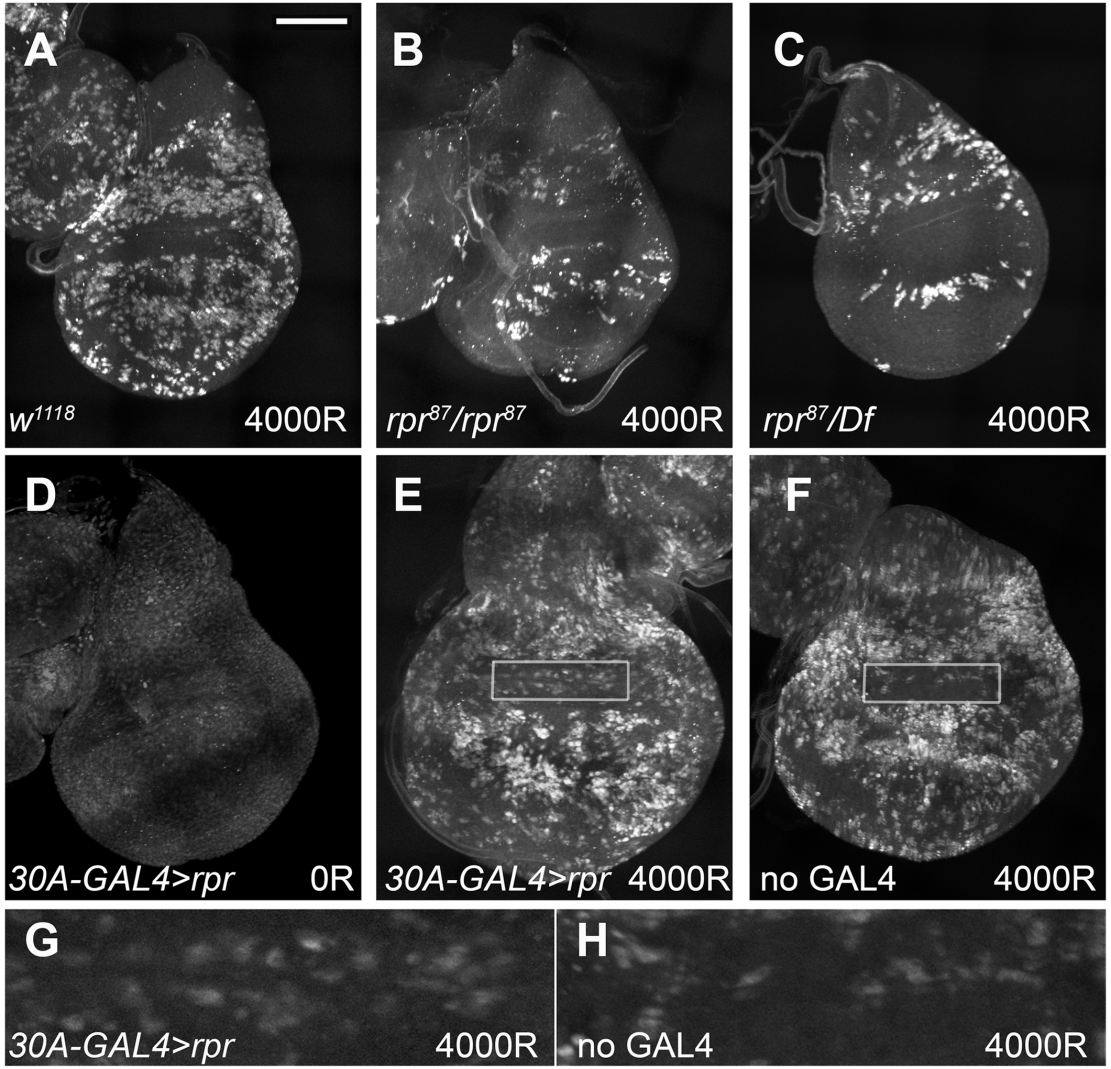
Alterations in *reaper* expression affect IR-induced apoptosis in the frown. Larval wing discs were fixed and stained for cleaved Dcp1. (A–C) Discs from 96–120 h old larvae 4 h after irradiation. *rpr* mutant discs show reduced IR-induced apoptosis compared to *w*^*1118*^ wild type controls. (D–H) Ectopic *rpr* promotes IR-induced apoptosis in the frown. 120–144 h old larvae raised at 20°C were irradiated with 0 or 4,000R of X-rays and shifted to 29°C immediately after irradiation to de-repress *rpr*. Larvae were dissected after 4 h at 29°C. Boxed sections of the frown in E and F are magnified 4X in G and H, respectively. *30A>rpr* = UAS-*rpr*/+; 30A-GAL4/+; GAL80^ts^/ GAL80^ts^. no GAL4 control = UAS-*rpr*/+; +/CyO-GFP; GAL80^ts^/ GAL80^ts^. Scale bar = 50 μm in A–F and 12.5 μm in G–H.

We further addressed the role of *rpr* in regulating apoptosis in the frown using gain- and loss-of-function experiments. *rpr*^*87*^ deletes -1860 to +661bp relative to *rpr* transcription start [32]. In larvae homozygous for *rpr*^*87*^ or trans-heterozygous for *rpr*^*87*^ and deficiency Df(3L)XR38, which removes *rpr* and other genes, IR-induced apoptosis was reduced throughout the wing disc (compare Fig 5A to 5B and 5C). These results demonstrate that reduction of *rpr* can prevent IR-induced apoptosis and support the idea that reduced *rpr* limits apoptosis in the frown. In reciprocal experiments, we induced UAS-*rpr*. After testing several GAL4 drivers, we identified *30A-GAL4* as specific to the hinge with little or no expression in the pouch (S6 Fig). De-repression of UAS-*rpr* using GAL80^ts^ and incubation at 29°C for 4 h did not induce apoptosis on its own (Fig 5D) but promoted IR-induced apoptosis in the frown (compare Fig 5E and 5G to the “no GAL4” sibling control in Fig 5F and 5H). These results also support the idea that limited *rpr* expression prevents IR-induced apoptosis in the frown.

### Cells of the Hinge Participate in Rebuilding the Pouch but Not Vice Versa

We next addressed the functional significance of apoptosis resistance in the future hinge cells of the wing disc. We hypothesized that these cells survive apoptosis-inducing insults in order to participate in subsequent tissue regeneration, much like stem cells. To test this hypothesis, we used a published lineage tracing system that relies on GAL4-driven expression of UAS-RFP to mark cells of interest [33]. Co-expression of UAS-FLP in these cells results in stable GFP expression by “flipping-out” intervening sequences, thus providing a clonal marker that persists even if the cells changed their identity and lost RFP expression.

**Fig 6:**
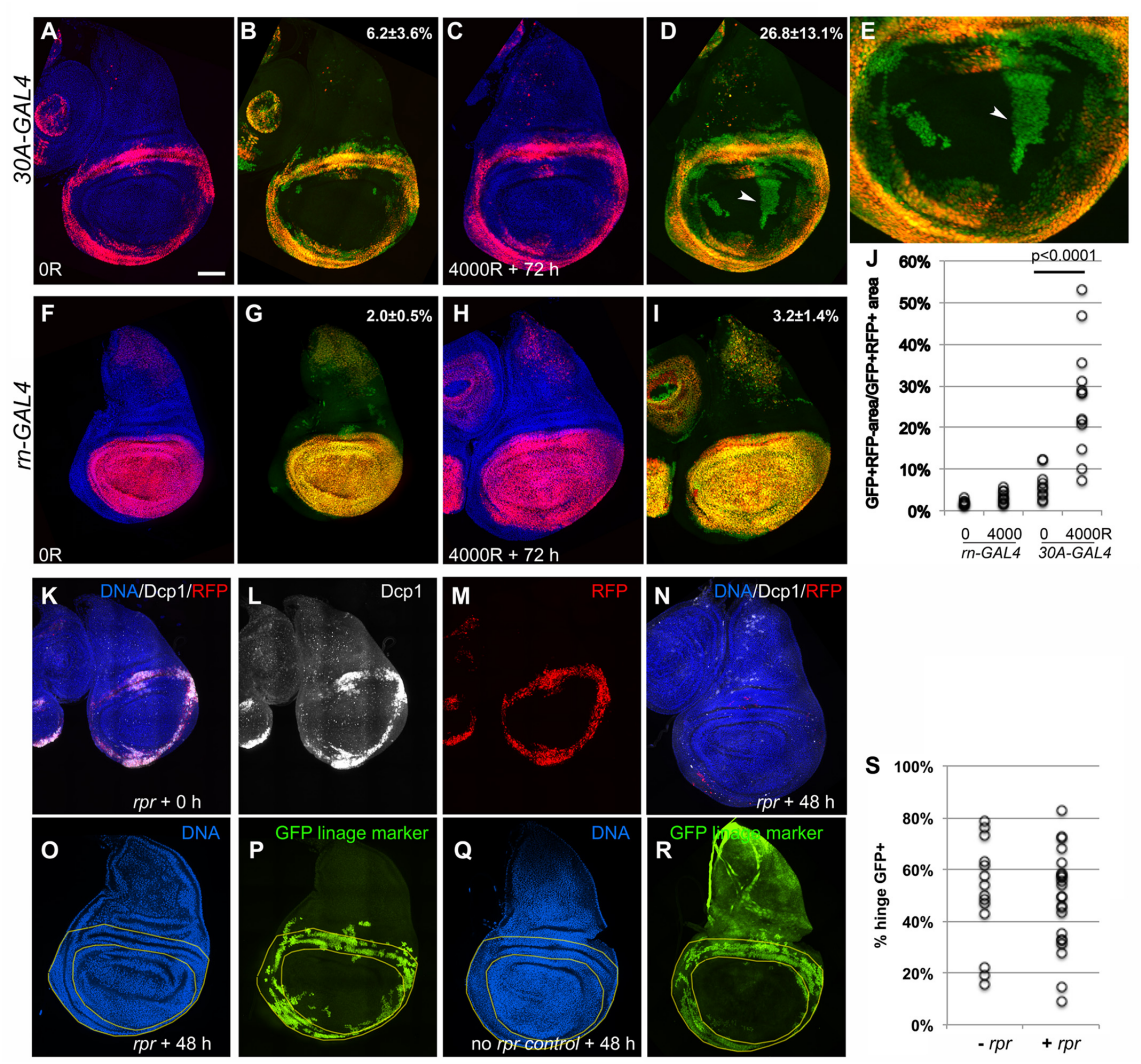
Cells of the hinge populate the pouch after irradiation. Embryos were collected at room temperature for 24 h. (A–J) Embryos were reared at room temperature for 72 h from the end of collection and shifted to 29°C for 24 h before irradiation. Larvae were then returned to 29°C to maintain Stringer expression for 72 h before dissection. For (K–S), embryos were reared at room temperature for 96 h from the end of collection, shifted to 29°C for 18 h to de-repress *rpr*, and returned to room temperature for 48 h before dissection. Rearing the larvae for 72 h instead of 96 h before *rpr* induction gave similar results. Wing discs were fixed and stained for DNA (blue) and imaged for RFP and GFP. Some of the discs were also stained for cleaved Dcp1 as indicated. (A,B) Wing discs from feeding third instar larvae of the genotype *30A-GAL4*/*Stringer* lineage-tracing chromosome (see Materials and Methods). RFP and GFP overlap in un-irradiated discs such that within the area enclosed by the ring of *30A-GAL4* expression, cells that have GFP but not RFP occupy an area that measures only 6.2±3.6% of the total area occupied by GFP+RFP+ cells. (C,D) Wing discs from larvae that were irradiated in feeding third instar stage. Many more cells that show GFP but not RFP are in the same area (arrowhead). Part of (D) is magnified in (E). (F–I) Wing discs from larvae of the genotype *Stringer*/+; *rn-GAL4*/+. GFP and RFP overlap in both un-irradiated (F,G) and irradiated discs (H,I) such that cells that have GFP but not RFP occupy less than 5% of the total area occupied by GFP+RFP+ cells. (J) The area occupied by GFP+RFP- cells is expressed as the total area occupied by GFP+RFP+ cells in each wing disc (the raw data can be found in the S1 Data file). In *rn-GAL4* samples, GFP+RFP+ area from the pouch region (i.e., not the notum) was used in the denominator, while GFP+RFP- area contiguous with the pouch area was used in the numerator. In *30A-GAL4* samples, GFP+RFP+ area from the hinge region was used in the denominator, while GFP+RFP- area enclosed by the above was used in the numerator. The *p*-value was calculated using a 2-tailed Student’s *t* test. (K–R) Wing discs from larvae of the genotype 30A>*Stringer*/+; *GAL80*^*ts*^*/GAL80*^*ts*^ with (K–P) and without (Q,R) one copy of *UAS-rpr*. (K–M) shows a disc immediately after induction of *rpr* for 18 h. Cleaved caspase signal (white in K,L) overlaps almost entirely with the 30A domain (red/RFP in K,M). After an additional 48 h, caspase signal and RFP are absent (N), but the hinge is populated with GFP+ cells that are from the 30A lineage (O,P). A control disc without *UAS-rpr* (Q,R) shows the expected 30A domain for larvae treated identically but without induction of cell death. Fractional area of the hinge (between the yellow lines) that is GFP+ was quantified from discs such as those in (P and R) and shown in S (the raw data can be found in the S1 Data file). Low level of GFP signal in the notum in (R) is from the CyO-GFP balancer. Scale bar = 25 μm in E and 50 μm in others.

Using the 30A-GAL4 driver described above, we saw coincidence of RFP and GFP in un-irradiated discs sampled at multiple points throughout feeding third larval instar stages (Fig 6A and 6B). Quantification of GFP+RFP- areas (cells that used to express RFP but have lost it) as a percent of total GFP+RFP+ area for each disc showed that cell fates were stable; less than 7% of hinge cells that ever expressed *30A-GAL4* became RFP- (Fig 6B and graphed in Fig 6J, “*30A-GAL4* 0R”). In contrast, in irradiated discs, GFP+RFP- cells were observed in the pouch 48 h after irradiation with 4,000–6,000R of X-rays. Quantification at 72 h after irradiation with 4,000R showed that GFP+RFP- cells occupied as much as 50% of the pouch in some discs, averaging ~27% (Fig 6C–6E, quantified in Fig 6J). Furthermore, the fact that migrant cells no longer expressed RFP meant that these cells not only relocated but also lost their original identity, such that *30A-GAL4* was no longer active.

Lack of cell death in the frown raised the possibility that cells with DNA damage are surviving in this region and contributing to regeneration. Published and new data help rule out this possibility. Prior analysis of the γ-H2Av, an indicator of DNA double strand breaks, showed that cells of the wing disc follow a similar time course of break and repair regardless of their location in the disc [34]. Briefly, robust induction of γ-H2Av was observed immediately after exposure to 4,000R of radiation but was reduced to background levels by 24 h after irradiation. We reproduced this result while also marking the hinge with 30A-GAL4>UAS-RFP; 24 h after irradiation with 4,000R, γ-H2Av signal was back to background levels throughout the disc (S7 Fig focuses on part of the frown and nearby pouch and notum cells). We conclude that repair of DNA breaks is complete before the movement of hinge cells into the pouch becomes detectable.

We note a published study that observed hinge cells invading the pouch [35]. A key difference in that study was the specific direction of apoptosis to the pouch by tissue-specific expression of *hid*, while hinge cells were deliberately kept alive. Here, we irradiated the entire disc, and indeed the entire larva, and allowed natural variations in apoptosis to occur. In this situation, cells of the hinge were protected (Figs 1–4) and became part of the pouch following irradiation.

To rule out the possibility that these results were due to IR-induced cell mixing within the wing disc, we performed a reciprocal experiment in which RFP/GFP markers were expressed using *rotund*-GAL4. *rn-GAL4* is active in the entire pouch and some inner hinge cells. Under similar experimental conditions as in *30A-GAL4* experiments, with or without irradiation, we saw near complete overlap of RFP and GFP (Fig 6F–6J). In other words, while irradiation resulted in hinge cells moving into the pouch, pouch cells did not move out to the hinge, ruling out general IR-induced cell mixing.

We also asked whether cells from outside the hinge are capable of rebuilding the hinge if cell death was directed to this area. We modified a published protocol by which transient expression of *hid* and an FLP recombinase-mediated lineage marker was directed to the pouch [35]. The authors found that the repaired pouch was made of only a small fraction (~25%) of lineage-marked cells, with the rest without the lineage marker, i.e., immigrant cells from outside the pouch. Here we directed the expression of *rpr* to the hinge using *30A-GAL4* while inducing *stringer-GFP* lineage marker at the same time. Induction of *rpr* for 18 h produced robust caspase cleavage in the 30A domain (Fig 6K–6M). Examination of wing discs 48 h from the end of *rpr/*GFP lineage marker induction showed a variable extent to which GFP+ cells occupy the hinge (Fig 6P, quantified in S as “+*rpr”*). The extent of GFP+ cells in the hinge was similar in control discs that also experienced an 18 h induction of the GFP lineage marker but without *rpr* (Fig 6R, “-*rpr”* in Fig 6S, *p*-value > 0.7; background GFP signal in the notum in R is from the CyO-GFP balancer that is absent in P). In other words, the composition of the hinge remained unchanged during recovery from *rpr*-induced cell death. These data support the idea that regeneration of the hinge relies mostly on GFP-marked hinge cells.

### Inhibition of STAT and Wg in the Future Hinge Interfered with Recovery after Irradiation

**Fig 7:**
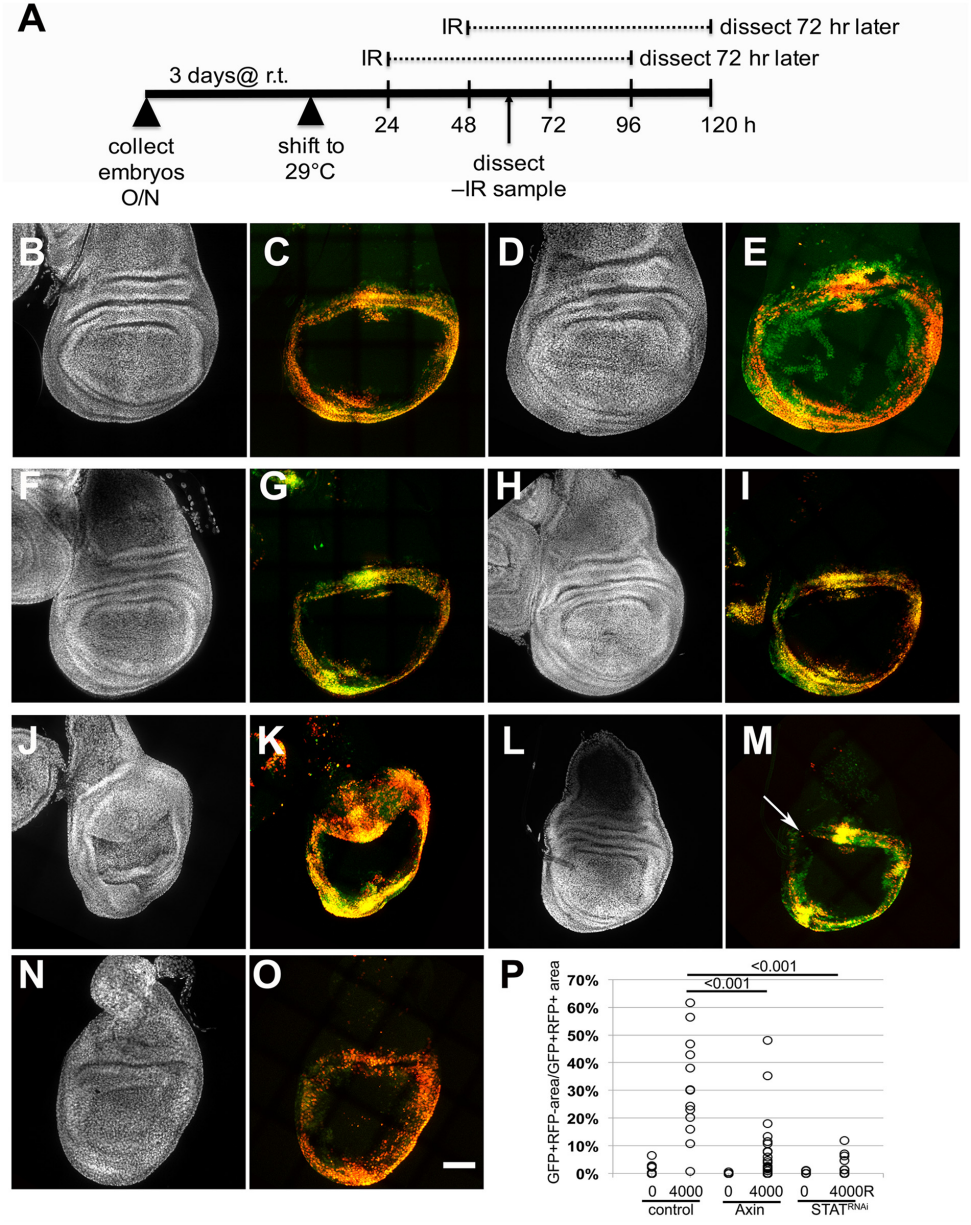
Inhibition of Wg and STAT interfered with cell relocation after irradiation. Wing discs were fixed and stained for DNA and imaged for RFP and GFP. (A) The experimental protocols used. r.t. = room temperature;–IR = 0R; and +IR = 4,000R of X-rays. Embryos were collected for 24 h at room temperature and aged for 72 h from the end of embryo collection. (B–O) Wing discs from third instar larvae of the genotype *30A-GAL4*>*Stringer* lineage-tracing chromosome/+; GAL80^ts^/+ (control); *30A-GAL4*>*Stringer* lineage-tracing chromosome/+; GAL80^ts^/UAS-Axin-GFP (Axin); or UAS-STAT^RNAi^/+; *30A-GAL4*>*Stringer* lineage-tracing chromosome/+; GAL80^ts^/+ (STAT). (P) The movement of GFP-RFP+ cells was quantified as in Fig 6J (the raw data can be found in the S1 Data file). The p-values were calculated using a 2-tailed Student’s *t* test. Scale bar = 50 μm.

Next, we expressed Axin or STAT^RNAi^ in the context of *30A-GAL4*-driven lineage markers to ask if Wg and STAT are needed for the relocation of cells to the pouch after irradiation. UAS-Axin or UAS-STAT^RNAi^ were induced by de-repression of GAL4 at 29°C for 24 or 48 h, with similar results (Fig 7A shows the experimental protocol). In control larvae without the UAS transgenes that were subjected to this protocol, wing discs appeared normal (Fig 7B–7E), and GFP+RFP- cells relocated into the area enclosed by the 30A-GAL4 domain after irradiation (Fig 7E; quantified in Fig 7P). Transient expression of Axin or STAT^RNAi^ under the control of *30A-GAL4* did not disrupt with GFP/RFP expression or wing morphology (S8 Fig). Thus, while Wg and STAT are required for wing development, their depletion in late third instar using the protocol shown in Fig 7A was apparently tolerated. The addition of irradiation, however, produced discs that were misshapen to varying degrees (e.g., Fig 7J and 7N) and showed interruption of the *30A* domain (arrow in Fig 7M). Nonetheless, we were able to quantify GFP+RFP- cells and found that induction of either Axin or STAT^RNAi^ reduced the extent to which such cells populated the pouch (Fig 7K, 7M, 7O and 7P). We conclude that Wg and STAT activities are required to rebuild the wing disc properly after irradiation.

## Discussion

We report four significant findings. First, we identified the frown, an IR-resistant domain of cells in the wing imaginal disc that participates in the rebuilding of the pouch after irradiation. Second, we found that both IR resistance and the ability to participate in regeneration are dependent on STAT and on Wg acting cell-autonomously. Third, we identified transcriptional repression of *rpr* as a potential mechanism by which Wg confers IR resistance in the frown. The fourth significant finding stems from the interpretation of the data in Fig 4 as follows. IR-induced apoptosis in *Drosophila*, as in other metazoans, is mediated, to a large extent, by p53. Dmp53 promotes apoptosis by transcriptional activation of pro-apoptotic genes such as *hid* and *rpr* [24,25]. This is recapitulated by the induction of the Hid-GFP reporter in a p53-dependent manner throughout the wing disc after exposure to IR ([31]; reproduced in Fig 4D). Based on this data, we infer that p53 is active in the cells of the frown following irradiation. However, it appears to be incapable of inducing in the same cells *rpr*, a known direct target of p53 [28], or apoptosis. This surprising result is not without precedent; ectopic and presumably homogeneous elevation of p53 using a heat-shock-inducible transgene induced apoptosis throughout the wing disc except in the frown [15]. In another example, adenovirus E4-ORF3 protein prevents the transcriptional activation of p53 target genes despite the presence of stable, active p53, by altering the chromatin structure at these loci [36]. We conclude that p53 activity in the frown, although capable of activating the *hid-GFP* promoter, is incapable of de-repressing *rpr*. Because ectopic *rpr* can induce apoptosis (Fig 4K) and promote IR-induced apoptosis (Fig 5), we suggest that the block in apoptosis occurs between p53 activation and *rpr* induction. Additional studies will be needed to understand how *rpr* is repressed while *hid* escapes this repression. A possible mechanism could be of epigenetic nature. In this regard, it is known that expression of *hid*, *rpr*, and *skl* are subject to epigenetic regulation through a cis-element called the irradiation responsive enhancer region (IRER) [37]. We note, however, that the IRER regulates both *hid* and *rpr*, whereas the mechanism we are interested in represses *rpr* but not *hid*.

STAT is clearly required to prevent IR-induced apoptosis in the frown, but the effect of STAT depletion on *rpr-lacZ* reporter expression was minimal. Instead, the published finding that DIAP1 is a direct transcriptional target of STAT [30] suggests another mechanism. This study found that the expression of a DIAP1 transcriptional reporter, especially in the hinge region throughout wing disc development, depended on STAT, especially in the hinge region. Thus, STAT activity may help maintain a threshold of DIAP1 level that Hid, Rpr, and Skl must collectively overcome. Such a threshold model, proposed by others before [26–29], could also explain why overexpressing *rpr* alone (e.g., Fig 4K) could induce robust apoptosis in the frown. On the other hand, repressing just one of these while the others respond normally to DNA damage appears to be sufficient to stop the cell from reaching the threshold necessary to overcome DIAP1 (e.g., *rpr* in Fig 5A–5C). Similarly, reducing Hid gene dosage by half in heterozygotes is sufficient to reduce and delay the onset of radiation-induced apoptosis [38]. So, in the frown, *hid* may be induced by IR, but the failure to induce *rpr* prevents these cells from ever reaching the threshold to undergo apoptosis. With reduced STAT activity, this threshold of DIAP1 may be lower, allowing Hid and Skl to overcome it. We also cannot rule out the possibility that STAT regulates yet another apoptosis-relevant target or acts redundantly with another regulator. The requirement for STAT is as strong as the requirement for Wg in the movement of hinge cells into the pouch after irradiation. We speculate that while Wg makes a greater contribution to protecting the frown cells from death (Axin had a stronger phenotype than STAT^RNAi^ in S4 Fig), both contribute equally to regeneration.

All the data reported here are from analyses of wing discs up to 3 d after irradiation, by which time the larvae had begun to pupariate. It is possible that the requirement for Wg and STAT are limited to these initial regenerative responses and that different regenerative mechanisms act later in development. If so, transient inhibition of Wg and STAT as we have done in Fig 7 may still be compatible with the production of relatively normal wings with the correct cell number. If, however, the period of regeneration is limited to within 3 d after irradiation, we would expect defective wings to result from inhibition of Wg or STAT during this time. Therefore, it would be informative in future studies to follow the development of wing discs into pupae and possibly into adult stages if they survive.

The data reported here indicate that STAT and Wg are necessary for radio-resistance of the frown. Are they sufficient? We think it unlikely for the following reasons. First, the D/V boundary expresses high Wg protein (e.g., Fig 2A), yet this region shows robust IR-induced cleaved caspase signal. As for STAT, a published study reported that induction of STAT in the eye disc resulted in cell-autonomous protection from IR-induced apoptosis [30]. Similar protection in wing discs was also reported in this study, but the data was not shown. In our reproduction of this experiment using the same GAL4 driver (*en-GAL4*) and the same UAS-STAT92E transgene, we saw little to no protection (S9 Fig). We propose that additional factors besides high STAT and Wg activity are required and that these are present in the frown but to a lesser extent elsewhere in the wing disc.

Gene expression characteristics in the frown that are relevant to IR resistance (e.g., low *rpr* expression) are already present before irradiation and maintained after irradiation. This suggests that cells within the frown are *intrinsically* resistant to apoptosis. This is in contrast to the phenomenon of *acquired* resistance to IR-induced apoptosis that we reported recently [9]. In what we named the Mahakali effect, dying cells in the wing disc protect nearby survivors from IR-induced apoptosis. The Mahakali effect requires *tie*, which encodes a receptor tyrosine kinase, and occurs through activation of an anti-apoptotic microRNA, *bantam*, in the protected cells. The intrinsic resistance (described here) and acquired resistance (published study) to IR-induced apoptosis are genetically separable; *tie* mutants that are defective for acquired resistance still show resistance in the frown [9].

The frown, although composed of columnar epithelial cells, is resistant to IR and participates in regeneration, much like stem cells are thought to do. In this regard, it is interesting that Wg and STAT are implicated in maintaining a *bona fide* stem cell population and promoting their proliferation when the need for regeneration arises. In the adult *Drosophila* gut, autocrine and paracrine signaling activate JAK/STAT, Wg and EGFR in intestinal stem cells (ISCs) to maintain the latter [39–42]. In response to tissue damage by chemicals or by microbial infection, additional signaling from damaged cells further activate the same pathways in ISCs to promote their proliferation and subsequent differentiation to replace lost cells [43–47]. Our results suggest an analogous situation in the wing disc but without the involvement of a dedicated stem cell population or a stem cell niche. The involvement of STAT we found in this mode of regeneration mirrors the results of two previous studies. STAT was activated in regenerating leg discs after surgical damage and in hemocytes in response to localized epidermal damage by ultraviolet radiation [48,49]. While the role of JAK/STAT activation in hemocytes was unclear, STAT activation in the leg discs was found to promote regenerative cell proliferation and to delay development.

There are other areas of the wing disc with high STAT or high Wg activity, yet resistance to IR-induced apoptosis is not as prominent or consistent in these areas as in the frown. Indeed, STAT must be active even in regions that show little or no reporter activity because STAT mutant clones in the pouch show phenotypes [17,30]. So, what distinguishes the frown from other regions of the wing disc? It lies between Wg inner ring and Wg outer ring. Wg-OR shows lower Wg expression than Wg-IR but is more robust along its dorsal boundary than in any other parts (e.g., [50]). Therefore, the frown in feeding third instar larvae could benefit not only from high STAT activity but also from two sources of Wg. We speculate that the combination of high STAT and possibly highest Wg signals makes the frown consistently resistant to apoptosis. This is in agreement with published studies that noted different adhesive and growth properties between cells of the hinge and the pouch, differences that appear to be related to Wg activity. For example, cells of the hinge show lower levels of E-Cadherin in adherens junctions, but elevation of Wg activity increased E-Cadherin levels, induced apical constrictions, and caused cells to bulge out, particularly in the hinge [51,52]. In another study, activation of Wg slowed proliferation in the pouch but accelerated proliferation in the hinge [53]. The accompanying report by W-M. Deng and colleagues shows that the same area also carries higher tumorigenic potential than the rest of the wing disc, further illustrating the special nature of this region [54].

The regulatory module we identified that operates through Wg, STAT, and Rpr, constitutes a new mechanism for resistance to IR-induced apoptosis. Through variations in gene expression/activity, a domain of resistant cells arises from among cells of identical genotype. A similar phenomenon has been described in mammalian cells in which, among a population of clonal cells, variations in the level or activity-state of proteins regulating receptor-mediated apoptosis can cause some cells to die while others survive exposure to the death-inducing ligand TRAIL [55]. Our work adds to this paradigm by showing that such cells and/or their clonal descendants can migrate, change fate, and participate in reconstruction of other tissue compartments.

Deregulation of Wnt and STAT pathways is associated with cancer. Wnt-1 (ortholog of Wg) was first identified as int1, a proto-oncogene targeted during viral carcinogenesis [56]. Likewise, persistent activation of STAT3 and, to a lesser extent, STAT5 is implicated in survival, proliferation, and invasion in cancer [57]. The prominence of STAT3 and STAT5 in cancer is interesting because the sole *Drosophila* STAT homolog is most like STAT3 and STAT5 in sequence and activity among the seven mammalian STATs [21]. More relevant to our study, deregulation of Wnt and STAT pathways is implicated in resistance to cytotoxic cancer therapy. Gene expression analysis across multiple cell lines found Wnt signaling and STAT3 among genes whose expression correlates with resistance to chemo-radiation or radiation [58,59] while combining radiation with a chemical inhibitor for STAT5 reduced survival in head and neck cancer cell lines [60]. Furthermore, inhibition of STAT3 reduced the ability of tumor-initiating colorectal carcinoma cells to form “tumor-spheres” in vitro [61], suggesting that STAT3 may be important for repopulation of tumors after treatment or for new metastases. Based on these considerations, we suggest that cells of the frown, which are protected from apoptosis by Wg and STAT and participate in tissue rebuilding after radiation damage, may provide a unique opportunity to study how these conserved signaling pathways ensure survival and regeneration in a genetically tractable model organism. For instance, we do not know the mechanism by which hinge cells relocate to the pouch. Directed cell divisions that “send” daughter cells into the pouch, cell migration, or a combination thereof, are some of the possible ways. Likewise, we also do not know the mechanisms by which hinge cells lose their hinge identity. We hope to exploit genetic tools to address these questions in the future.

## Materials and Methods

### Fly Stocks

These stocks are described in Flybase: *w*^*1118*^, *y*^*1*^*w*^*1118*^, *wg*^*I-12*^, *wg*^*I-16*^ *(Df(2L)wg-CX3, wg[l-16] b[1] pr[1]/CyO)*, *STAT92E*^*F*^, *STAT92E*^*06346*^, *ptc-GAL4* (on Ch II), *en-GAL4* (on Ch II), *rn-GAL4* (on Ch III), *30A- GAL4* (on Ch II, Bloomington stock#37534), *Ptub-GAL80*^*ts*^ on Ch III, 10XSTAT-GFP, *UAS-GFP* (on Ch II), *UAS-TCF*^*DN*^ (on Ch III), *UAS-Axin-GFP* (on Ch III), *UAS-STAT*^*RNAi*^ (on X), *rpr-11-lacZ* (on Ch III). The stock used for lineage tracing is also described in Flybase; w*; P{UAS-RedStinger}4, P{UAS-FLP.D}JD1, P{Ubi-p63E(FRT.STOP)Stinger}9F6 /CyO (Bloomington stock#28280). *STAT92E*^*F*^ is a temperature-sensitive EMS-induced point mutation [62]. *STAT92E*^*06346*^ is a strong loss-of-function allele caused by a transposon-insertion that produces no transcript or protein [63,64]. *wg*^*1-12*^ is an EMS-induced temperature sensitive allele [22].

Other stocks: *DIAP1-lacZ* [65], *hid-GFP* [31], ci-GAL4 [66], *UAS-rpr* (on X, [67]), UAS-STAT92E [30], *rpr*^*87*^ [32], and Df(3L)XR38 [68].

### Larvae Culture

Larvae were raised on Nutri-Fly Bloomington Formula food (Genesee Scientific) at 25°C unless otherwise noted. The cultures were monitored daily for signs of crowding, typically seen as “dimples” in the food surface as larvae try to increase the surface area for access to air. Cultures were split at the first sign of crowding. Larval timing, i.e., that we were working with feeding stage three instar larvae, was through a combination of larval age and size (late third instar larvae were significantly bigger than second instar in the absence of crowding) and their location in the food.

### Irradiation and Drug Treatment

Larvae in food were irradiated in a Faxitron Cabinet X-ray System Model RX-650 (Lincolnshire, IL) at 115 kv and 5.33 rad/s. For maytansinol treatment, larvae at 94–98 h after egg deposition were transferred to food containing 2 μM maytansinol or DMSO control. Wing discs were dissected 24 h after the transfer.

### Antibody Staining and TUNEL

Cleaved Caspase 3 (Drice) (1:100, rabbit polyclonal, Cell Signaling Cat# 3661) was used as described before [38]. Antibodies to cleaved Dcp1 (1:100, rabbit polyclonal, Cell Signaling Cat# 9578) Phospho-Histone H3 (1:1000, rabbit monoclonal, Upstate Biotech), Wingless (1:100, mouse monoclonal, Drosophila Hybridoma Bank Cat# 4D4), β-galactosidase (1:100, Developmental Hybridoma Bank Cat#40-1a) were used as described before [25]. Anti-γ-H2Av antibody (1:2000, mouse monoclonal, Drosophila Hybridoma Bank Cat# UNC93-5.2.1) was used as described before [69]. Secondary antibodies were used at 1:100 (Jackson). For antibody staining, wing discs were dissected in PBS, fixed in 4% para-formaldehyde in PBS for 30 min, and washed three times in PBS, permeabilized in PBTx (0.5% Triton X-100) for 10’, and rinsed in PBTx (0.1% Triton X-100). The discs were blocked in 5% Normal Goal Serum in PBTx (0.1% Triton X-100) for at least 30 min and incubated overnight at 4°C in primary antibody in block. The discs were rinsed thrice in PBTx (0.1% Triton X-100) and incubated in secondary antibody in block for 2 h at room temperature. For TUNEL, wing discs were dissected in PBS, fixed in 4% para-formaldehyde in PBS for 20 min, and washed three times in 0.3% Triton X-100/PBS for at least 20 min total. The discs were permeabilized overnight at 4°C in 0.3% Triton X-100/PBS, followed by three washes in 0.3% Triton X-100/PBS for at least 10 min total. The discs were processed using an Apoptag Red kit (Millipore), according to the manufacturer’s instructions.

Stained discs were washed in PBT. The discs were counter-stained with 10 ug/ml Hoechst33258 in PBT or PBTx (0.1%TritonX-100) for 2 min, washed three times, and mounted on glass slides in Fluoromount G (SouthernBiotech).

### EdU Labeling

Wing discs were dissected from wild-type (*y*^*1*^*w*^*1118*^) or STAT-GFP reporter larvae, incubated in 10 μM EdU in Schneider’s Insect Medium (Sigma) for 1 hr, and processed to detect incorporated EdU according to the manufacturer’s instructions (Alexa647 Click-iT Edu Imaging Kit, Molecular Probes).

### In Situ Hybridization for *hid* mRNA

A cocktail of 48 Quasar 570-labeled custom-made anti-sense probes from Stellaris_LGC Biosearch Technologies were used. Probe sequences in the 5′–3′ direction as generated by Stellaris probe designer version 4.1 against *hid* mRNA variant B (GenBank Accession NM_001275081) were:

1gctccgcggctaaaaatgaa; 2agggcaggaaacacgtctta; 3acgtgtcgcagactcaaaga; 4tttgcttttgctgttgtcaa; 5actgttcacgatggatttcg; 6acccttttcgtgtttagaac; 7gattcttctgcgtttttcat; 8caagtttttgctcggttagt; 9ttatctttcctgatttgtca; 10tttttcgtgcagttttttct; 11gattttgtatttcttgtgca; 12acttttggttagagttcact; 13gctttgttttgctttttatt; 14cttcttgtgattgttcttcg; 15tgcactttgttggcactttg; 16ggcaaataaaagggcacggc; 17gatgaactcgacgctacgtc; 18aggaggagacggacgaggat; 19cgatgcggaggacgaagatg; 20tagagggcgtatagcacttg; 21cggcggatactggaagattt; 22cgtgaaattgcaagaggggc; 23ccgtgcggaaagaacacatc; 24attcgagttcggattcggat; 25ggaagaagttgtactcctcg; 26gatatgacggatgtggttgc; 27gaatggtgtggcatcatgtg; 28tcatgatcgctctggtactc; 29aaagttgtcgtagcgatcgc; 30tccattgaactcctgcagac; 31tattggagctcttcttcttc; 32ggtatggcagactggattat; 33gactgatgtggccatggatg; 34tctgtggtttcttcttctcg; 35acaacagttggccaagtgaa; 36gcccatggccaaaacgaaaa; 37ttcatcgcgccgcaaagaag; 38attcgattacacgtctcctg; 39cttaagggctagctgatttc; 40aactatgtttagatcggca; 41gttgcacttatgtacggttt; 42cgctcctgcagttcaataaa; 43atgttggctgtttgtgtatc; 44ccttcttaatcttaggcaca; 45atatattgttcttgtgtccc; 46tgcagttaccatagacagat; 47gttatctttcgtttcgtttt; 48cttgccagtctaagagtttt.

90–102 h old larvae were dissected in PBS 2 h after irradiation and fixed in 4% paraformaldehyde in PBS for 30 min at room temperature. The discs were dehydrated for 24 h at 4°C in 70% ethanol. The samples were hybridized with 0.05 μM probe for 16 h at 37°C in the dark. All other experimental conditions were as published [70]. The discs were counter-stained with 5 μg/ml Hoechst33258 for DNA. The samples were mounted in 50% glycerol, 150 mM NaCl, and 15 mM sodium citrate for imaging.

### Image Analysis

With the exceptions noted below, the discs were imaged on a Perkin Elmers spinning disc confocal attached to a Nikon inverted microscope, using a SDC Andor iXon Ultra (DU-897) EM CCD camera. The NIS- Elements acquisition software’s large image stitching tool was used for the image capture. Twenty to 21 Z-sections 1 um apart were collected per disc and collapsed using “maximum projection” in Image J. The exceptions are: Fig 4A–4G were acquired on a Nikon inverted microscope with a Hamamatsu image EM C9 100–13 EM CCD camera; Fig 4A and 4B show a single optical section each; Fig 4C and 4D were optical sections collapsed using “sum projection” in image J; S1F and S1G Fig were imaged on a Q-Imaging R6 CCD camera using Ocular software.

## Supporting Information

S1 Data Excel spreadsheet containing, in separate sheets, the underlying numerical data and statistical analysis for Figs 6J, 6S and 7P. (XLSX)

S1 Fig Resistance to apoptosis varies within a larval wing imaginal disc. (A, B) Wing discs were dissected from 92 to100 h old feeding third instar wild-type (*y*^*1*^*w*^*1118*^) larvae 4 h after exposure to 4000R of X-rays, fixed and stained with an antibody to cleaved Caspase 3 (A, red) or processed for TUNEL assay (B, red). The discs were also stained for DNA (blue). (C) Wing discs from feeding wild-type (*w*^*1118*^) larvae that were fed food containing 0.5 μM maytansinol for 24 h, from 96 to 120 h after egg laying. The discs were fixed and stained with an antibody to cleaved caspase Dcp1 (red) and DNA (blue). (D–E) A montage of Z-sections for the disc in Fig 1D. Fig 1D is a projection of Z-sections collected 1 micron apart, but only every other section is shown here for DNA (D) and Dcp1 (E), starting with the most apical (peripodial) layer. (F–G) Wing discs stained for DNA (F) and incorporated EdU (G) that indicates cells in S phase. Larvae were 96–120 h old at the time of exposure to EdU for 1 hr. Images are shown with anterior left and dorsal up. Scale bar = 50 μm.
(TIF)

S2 Fig STAT and Wg are not required for the expression of each other. Wing discs were dissected from feeding third instar larvae 4 h after irradiation with 0 or 4,000R, fixed and stained for DNA or Wg. Images are shown with anterior left and dorsal up. (A) A schematic representation of the Wg signaling pathway in *Drosophila* (modified from [71]). Components manipulated in this study are shaded: Wg, Axin, and TCF. TCF is a transcription factor. Axin promotes the APC/proteasome-mediated degradation of signal transducer Armadillo (β-catenin). (B, C) *ci*-GAL4/STAT-GFP; UAS-TCF^DN^/GAL80^ts^ transgenic embryos were collected at 18°C for 24 h, reared at 18°C for 6 d, and shifted to 29°C for 48 h to inactivate GAL80^ts^ and induce UAS-TCF^DN^ expression, before irradiation. Wing discs were imaged for GFP (green) and DNA (blue). A and P compartments were not marked in these larvae, so the anterior/posterior boundary (white line) is approximate. UAS-TCF^DN^ did not alter STAT-GFP expression, with or without irradiation; there was little difference in STAT-GFP between A and P halves of the frown. (D, E) Wing discs from *STAT92E*^*F*^*/STAT92E*^*06346*^ trans-heterozygous larvae, stained with an antibody to Wg. Embryos were collected at 18°C for 24 h, reared at 18°C for 5 d, and shifted to 29°C for 72 h to inactivate temperature-sensitive *STAT92E*^*F*^ before irradiation. Rearing larvae for 6 d at 18°C and shifting to 29°C for 48 h produced similar results. Wg expression was robust in STAT mutants after the temperature shift, and no further changes after irradiation. Scale bar = 50 μm.
(TIF)

S3 Fig Expression of TCF^DN^ results in IR-induced apoptosis in the *frown*. (A–D) *en*-GAL4/+; UAS-TCF^DN^/GAL80^ts^ transgenic embryos were collected for 24 h at 18°C, raised at 18°C for 7 d, and shifted to 29°C for 24 h to inactivate GAL80^ts^ before exposure to 0 or 4,000R of X-rays. Wing discs were dissected 4 h after irradiation, fixed and stained for cleaved Dcp1 and for DNA. TCF^DN^ expression in the posterior compartment increased the nuclear density (A) and caused spontaneous apoptosis in the pouch (B). The pouch/hinge regions in (C) are magnified in (D). Anterior/posterior boundary, discerned using UAS-GFP expressions, is marked with white lines. The frown is between yellow lines. Arrow indicates increased apoptosis in the posterior half of the frown where cells were expressing TCF^DN^ compared to the control anterior half. Scale bar = 50 μm in A–C and 25 μm in D.
(TIF)

S4 Fig The effect of co-depletion of STAT and Wg on cell death in the frown. Embryos were collected at 18°C for 24 h, reared at 18°C for 6 d, and shifted to 29°C for 48 h before irradiation with 0 or 4,000R. Wing discs were dissected from feeding third instar larvae 4 h after irradiation, fixed and stained with an antibody to cleaved caspase Dcp1 and for DNA. The DNA stain was used to locate the frown (between yellow lines) as two folds of cells (see Fig 2I). GFP expression was used to discern the A/P boundary (white lines). All larvae were age-matched siblings from the same cross. GAL4 was de-repressed by incubation at 29°C for 48 h immediately before irradiation. Images are shown anterior left and dorsal up. (A-C) *UAS-STAT*^*RNAi*^*/+; en-GAL4>UAS-GFP/+; GAL80*^*ts*^*/+*. There is more Dcp1 signal in the P half of the frown (arrow) than in the A half. (D, F) *+/Y; en-GAL4>UAS-GFP/+; UAS-Axin-GFP/+; GAL80*^*ts*^*/+*. There is more Dcp1 signal in the P half of the frown (arrow) than in the A half. (G-L) *UAS-STAT*^*RNAi*^*/+; en-GAL4>UAS-GFP/+; UAS-Axin-GFP/ GAL80*^*ts*^. Axin and STAT^RNAi^ did not induce apoptosis in the frown in un-irradiated controls (arrow in H) but did in irradiated discs (arrow in K). Scale bar = 50 μm.
(TIFF)

S5 Fig *rpr-lacZ* transcriptional reporter in irradiated *wg*^*I-12*^*/wg*^*I-12*^ discs *wg*^*I-12*^*/wg*^*I-12*^ mutants carrying a copy of the *rpr-lacZ* reporter were cultured as described for *wg*^*I-12*^*/wg*^*I-12*^ mutants in Fig 3L–3N. Wing discs were fixed and stained for DNA and for β-galactosidase 4 h after exposure to 4000R of X-rays. Scale bar = 50 μm.
(TIF)

S6 Fig The location of the *30A-GAL4* domain. Wing discs were dissected from feeding third instar larvae, fixed, and stained with an antibody for Wg protein (green) and for DNA (blue). *30A-GAL4* drives the expression of RFP (red). Wg Inner Ring (arrow) and outer ring (arrowhead) are indicated. The pouch is the inner-most “circle” within the Wg inner ring (see Fig 1b in [53]) and is indicated with dashed lines. Note the absence of RFP+ cells in the pouch. Scale bar = 50 μm. Embryo collection and larval culture were as in Fig 5.
(TIF)

S7 Fig The time course of γ-H2Av staining in the frown. Ninety-two to 100 h old feeding third instar larvae of the genotype *30A-GAL4*/*Stringer* lineage-tracing chromosome (see Materials and Methods) were irradiated with 0 or 4,000R of X-rays. Wing discs were dissected at time points shown, fixed, and stained with an antibody for γ-H2Av (gray) and DNA (blue). The discs were also imaged for RFP that mark the hinge (red). The panels focus on the dorsal hinge frown region. Scale bar = 5 μm.
(TIF)

S8 Fig 30A expression domain and the hinge appears normal in STAT^RNAi^ and Axin expressing wing discs at the time of irradiation. Wing discs were fixed and stained for DNA and imaged for RFP and GFP. The experimental protocol was as in Fig 7A. Larvae were dissected at 24 and 48 h after shift to 29°C, i.e., at the time of irradiation (IR). (A–D) Wing discs from third instar larvae of the genotype UAS-STAT^RNAi^/+; *30A-GAL4*>*Stringer* lineage-tracing chromosome/+; GAL80^ts^/+. (A, B) 24 h time point; (C, D) 48 h time point. (E–H) Wing discs from third instar larvae of the genotype *30A-GAL4*>*Stringer* lineage-tracing chromosome/+; GAL80^ts^/UAS-Axin-GFP. (E, F) 24 h time point; (G, H) 48 h time point. Scale bar = 50 μm.
(TIF)

S9 Fig Ectopic expression of STAT has little effect on IR-induced apoptosis. Embryos were collected at 25°C for 8–12 h, reared at 25°C for 96 h from the end of collection, and shifted to 29°C for 24 h to de-repress GAL4 before irradiation with 4,000R of X-rays. Wing discs were dissected 4 h later, fixed and stained for cleaved caspase Dcp1 and DNA, and imaged also for GFP. (A, B) Wing disc from control larvae expressing GFP in the posterior compartment. (C, D) Wing disc from larvae expressing GFP and STAT92E in the posterior compartment. Scale bar = 50 μm.
(TIF)

## Abbreviations

DIAP1: *Drosophila* inhibitors of apoptosis proteins 1
IR: ionizing radiation
IRER: irradiation responsive enhancer region
STAT: signal transducer and activator of transcription
Wg: Wingless
