# Supplementary figures and images for: Drosophila Wnt and STAT Define Apoptosis-Resistant Epithelial Cells for Tissue Regeneration after Irradiation
Source: PLoS Biol. 2016 Sep 1;14(9):e1002536. doi: 10.1371/journal.pbio.1002536 (PMC5008734; doi:10.1371/journal.pbio.1002536)

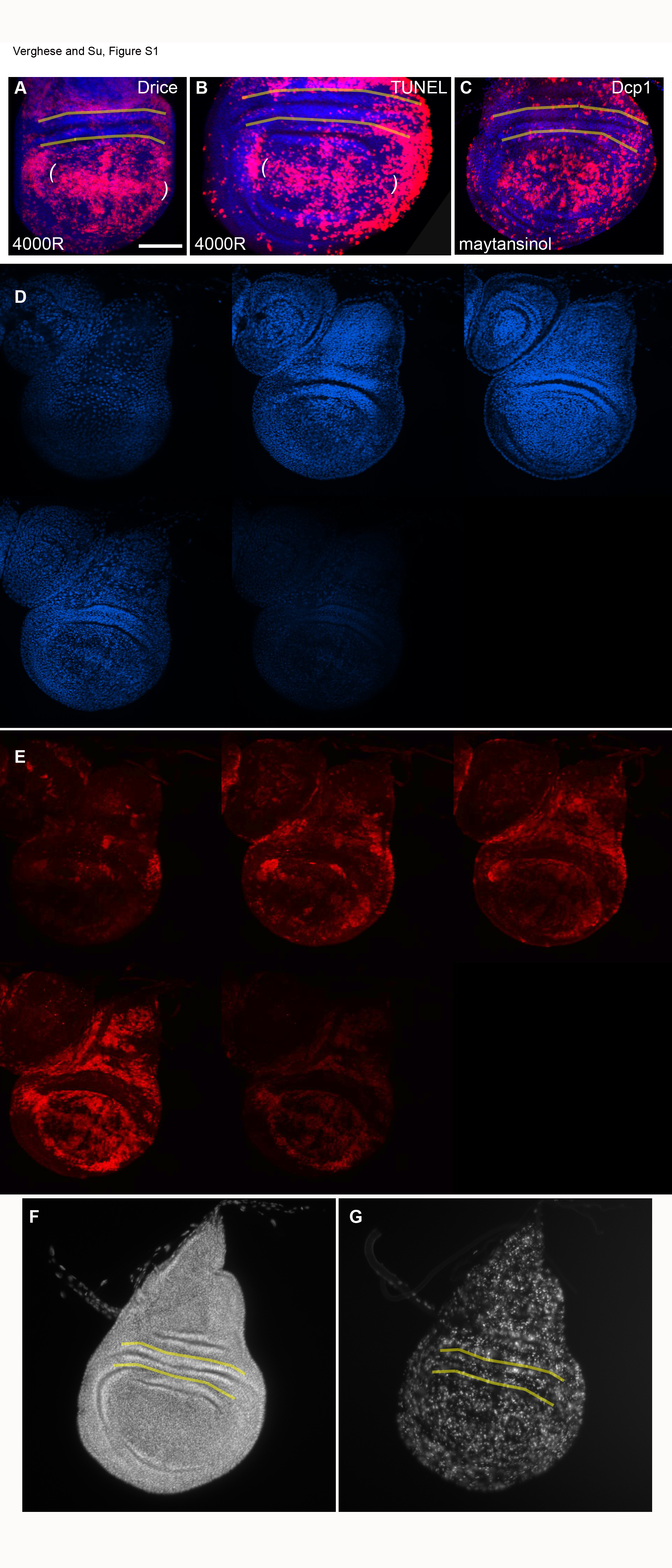

Supplement: S1 Fig — (A, B) Wing discs were dissected from 92 to100 h old feeding third instar wild-type (y1w1118) larvae 4 h after exposure to 4000R of X-rays, fixed and stained with an antibody to cleaved Caspase 3 (A, red) or processed for TUNEL assay (B, red). The discs were also stained for DNA (blue). (C) Wing discs from feeding wild-type (w1118) larvae that were fed food containing 0.5 μM maytansinol for 24 h, from 96 to 120 h after egg laying. The discs were fixed and stained with an antibody to cleaved caspase Dcp1 (red) and DNA (blue). (D–E) A montage of Z-sections for the disc in Fig 1D. Fig 1D is a projection of Z-sections collected 1 micron apart, but only every other section is shown here for DNA (D) and Dcp1 (E), starting with the most apical (peripodial) layer. (F–G) Wing discs stained for DNA (F) and incorporated EdU (G) that indicates cells in S phase. Larvae were 96–120 h old at the time of exposure to EdU for 1 hr. Images are shown with anterior left and dorsal up. Scale bar = 50 μm. (TIF) [file pbio.1002536.s002.tif]

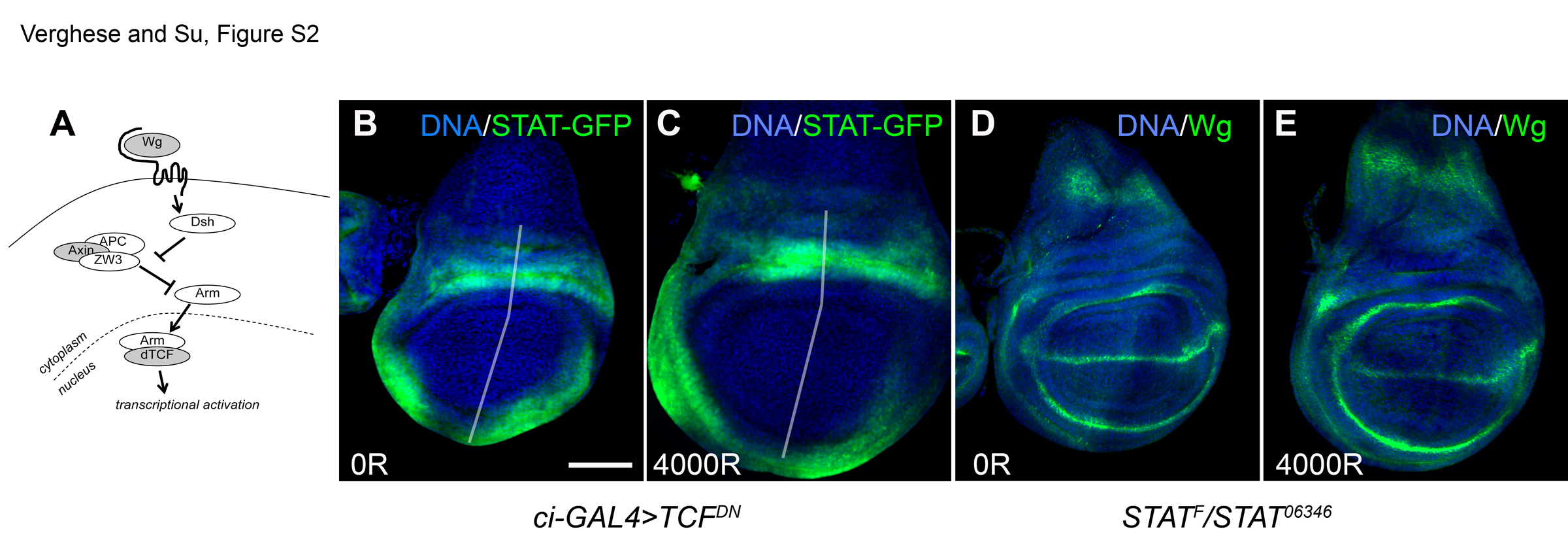

Supplement: S2 Fig — Wing discs were dissected from feeding third instar larvae 4 h after irradiation with 0 or 4,000R, fixed and stained for DNA or Wg. Images are shown with anterior left and dorsal up. (A) A schematic representation of the Wg signaling pathway in Drosophila (modified from [71]). Components manipulated in this study are shaded: Wg, Axin, and TCF. TCF is a transcription factor. Axin promotes the APC/proteasome-mediated degradation of signal transducer Armadillo (β-catenin). (B, C) ci-GAL4/STAT-GFP; UAS-TCFDN/GAL80ts transgenic embryos were collected at 18°C for 24 h, reared at 18°C for 6 d, and shifted to 29°C for 48 h to inactivate GAL80ts and induce UAS-TCFDN expression, before irradiation. Wing discs were imaged for GFP (green) and DNA (blue). A and P compartments were not marked in these larvae, so the anterior/posterior boundary (white line) is approximate. UAS-TCFDN did not alter STAT-GFP expression, with or without irradiation; there was little difference in STAT-GFP between A and P halves of the frown. (D, E) Wing discs from STAT92EF/STAT92E06346 trans-heterozygous larvae, stained with an antibody to Wg. Embryos were collected at 18°C for 24 h, reared at 18°C for 5 d, and shifted to 29°C for 72 h to inactivate temperature-sensitive STAT92EF before irradiation. Rearing larvae for 6 d at 18°C and shifting to 29°C for 48 h produced similar results. Wg expression was robust in STAT mutants after the temperature shift, and no further changes after irradiation. Scale bar = 50 μm. (TIF) [file pbio.1002536.s003.tif]

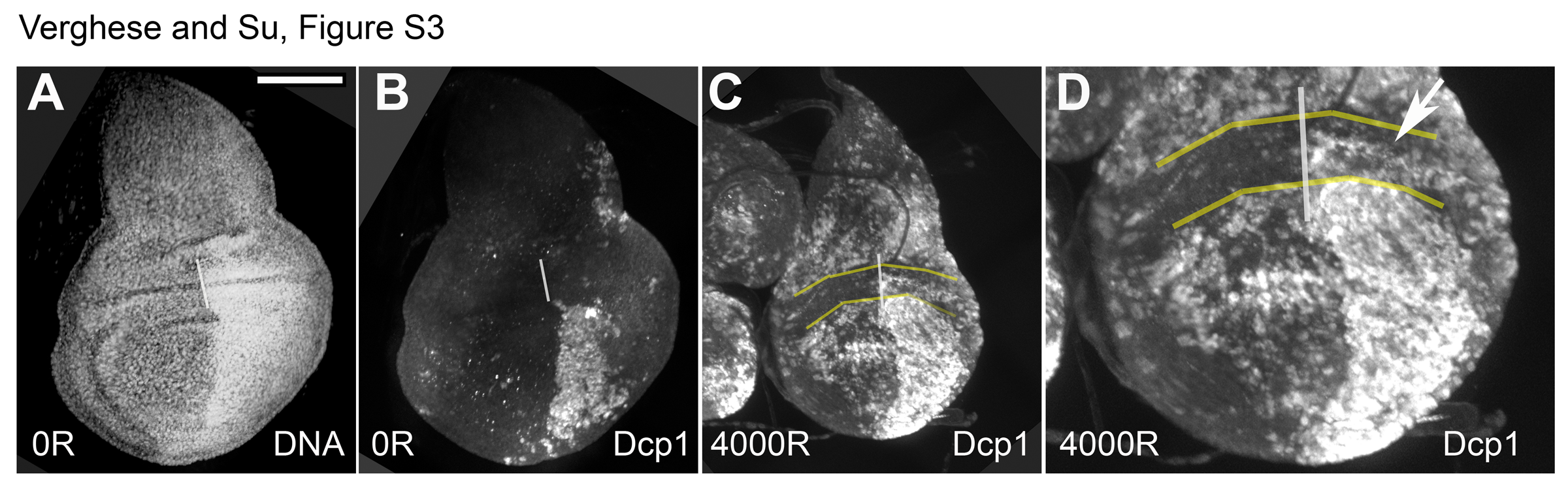

Supplement: S3 Fig — (A–D) en-GAL4/+; UAS-TCFDN/GAL80ts transgenic embryos were collected for 24 h at 18°C, raised at 18°C for 7 d, and shifted to 29°C for 24 h to inactivate GAL80ts before exposure to 0 or 4,000R of X-rays. Wing discs were dissected 4 h after irradiation, fixed and stained for cleaved Dcp1 and for DNA. TCFDN expression in the posterior compartment increased the nuclear density (A) and caused spontaneous apoptosis in the pouch (B). The pouch/hinge regions in (C) are magnified in (D). Anterior/posterior boundary, discerned using UAS-GFP expressions, is marked with white lines. The frown is between yellow lines. Arrow indicates increased apoptosis in the posterior half of the frown where cells were expressing TCFDN compared to the control anterior half. Scale bar = 50 μm in A–C and 25 μm in D. (TIF) [file pbio.1002536.s004.tif]

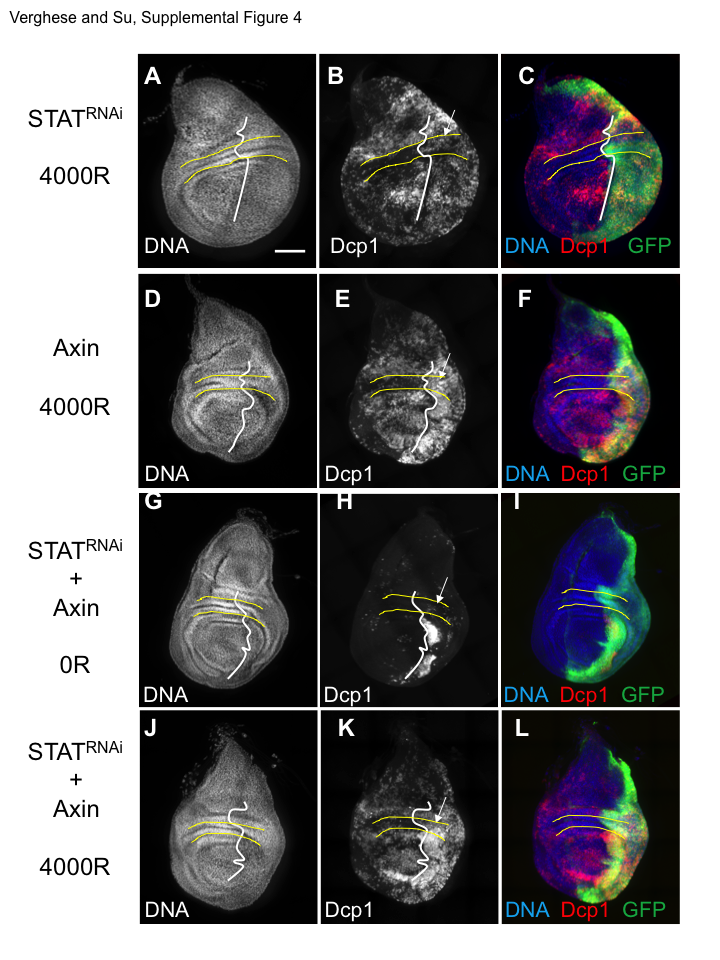

Supplement: S4 Fig — Embryos were collected at 18°C for 24 h, reared at 18°C for 6 d, and shifted to 29°C for 48 h before irradiation with 0 or 4,000R. Wing discs were dissected from feeding third instar larvae 4 h after irradiation, fixed and stained with an antibody to cleaved caspase Dcp1 and for DNA. The DNA stain was used to locate the frown (between yellow lines) as two folds of cells (see Fig 2I). GFP expression was used to discern the A/P boundary (white lines). All larvae were age-matched siblings from the same cross. GAL4 was de-repressed by incubation at 29°C for 48 h immediately before irradiation. Images are shown anterior left and dorsal up. (A-C) UAS-STATRNAi/+; en-GAL4>UAS-GFP/+; GAL80ts/+. There is more Dcp1 signal in the P half of the frown (arrow) than in the A half. (D, F) +/Y; en-GAL4>UAS-GFP/+; UAS-Axin-GFP/+; GAL80ts/+. There is more Dcp1 signal in the P half of the frown (arrow) than in the A half. (G-L) UAS-STATRNAi/+; en-GAL4>UAS-GFP/+; UAS-Axin-GFP/ GAL80ts. Axin and STATRNAi did not induce apoptosis in the frown in un-irradiated controls (arrow in H) but did in irradiated discs (arrow in K). Scale bar = 50 μm. (TIFF) [file pbio.1002536.s005.tiff]

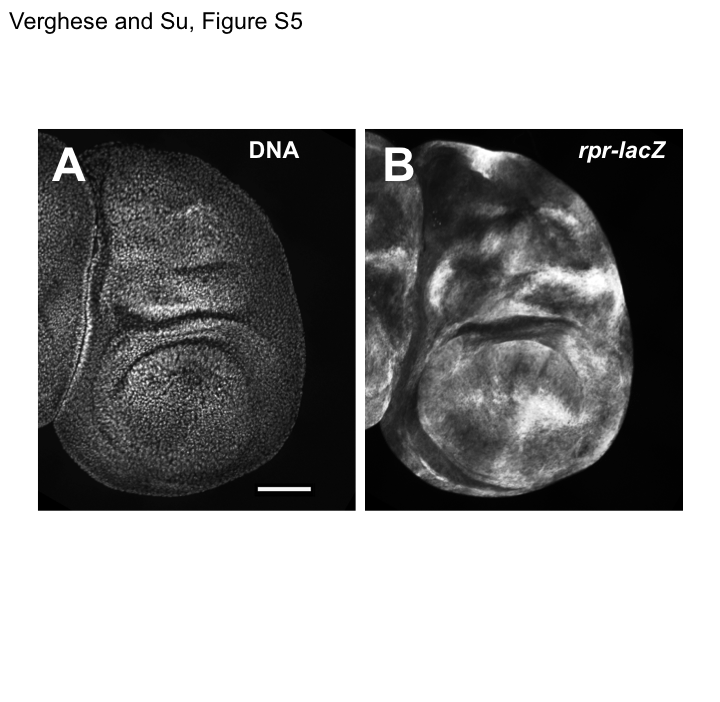

Supplement: S5 Fig — wgI-12/wgI-12 mutants carrying a copy of the rpr-lacZ reporter were cultured as described for wgI-12/wgI-12 mutants in Fig 3L–3N. Wing discs were fixed and stained for DNA and for β-galactosidase 4 h after exposure to 4000R of X-rays. Scale bar = 50 μm. (TIF) [file pbio.1002536.s006.tif]

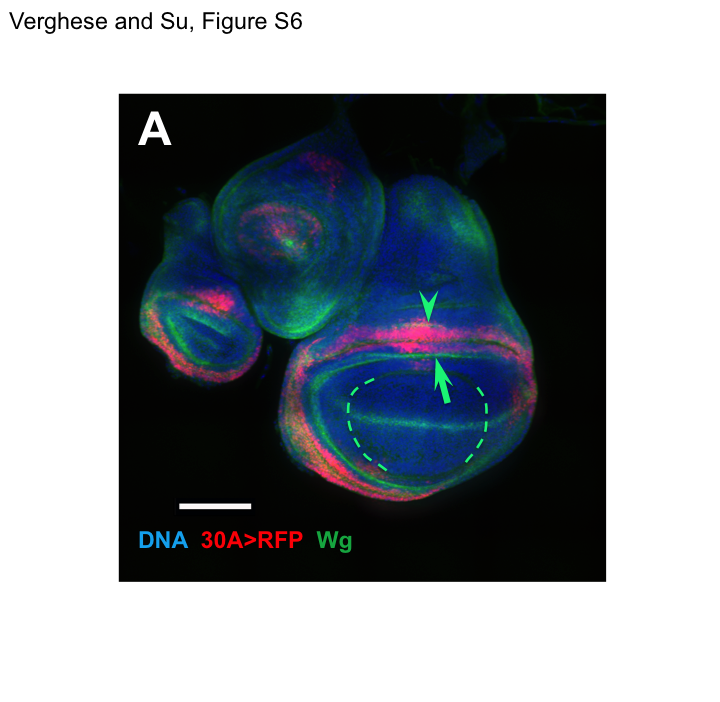

Supplement: S6 Fig — Wing discs were dissected from feeding third instar larvae, fixed, and stained with an antibody for Wg protein (green) and for DNA (blue). 30A-GAL4 drives the expression of RFP (red). Wg Inner Ring (arrow) and outer ring (arrowhead) are indicated. The pouch is the inner-most “circle” within the Wg inner ring (see Fig 1b in [53]) and is indicated with dashed lines. Note the absence of RFP+ cells in the pouch. Scale bar = 50 μm. Embryo collection and larval culture were as in Fig 5. (TIF) [file pbio.1002536.s007.tif]

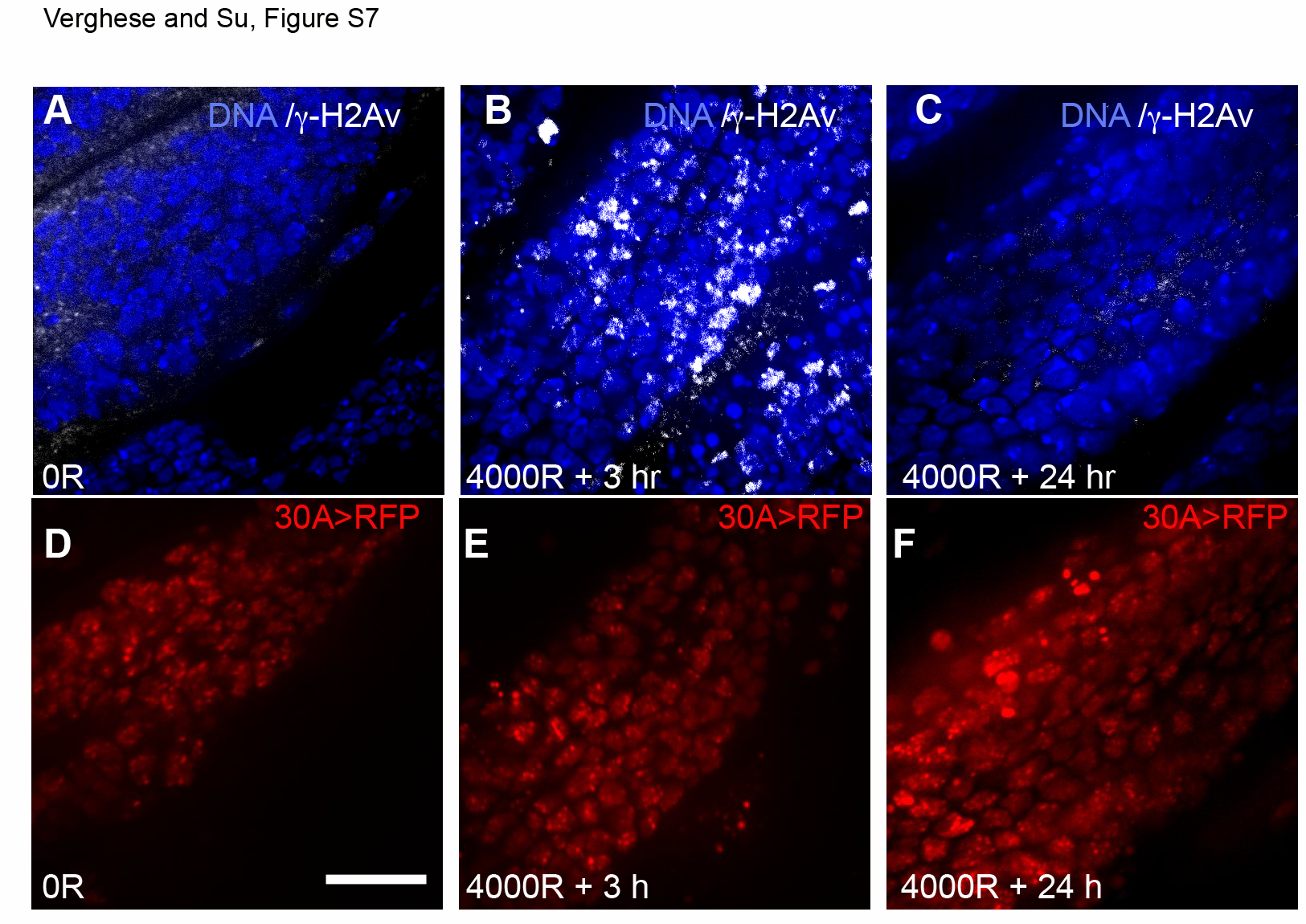

Supplement: S7 Fig — Ninety-two to 100 h old feeding third instar larvae of the genotype 30A-GAL4/Stringer lineage-tracing chromosome (see Materials and Methods) were irradiated with 0 or 4,000R of X-rays. Wing discs were dissected at time points shown, fixed, and stained with an antibody for γ-H2Av (gray) and DNA (blue). The discs were also imaged for RFP that mark the hinge (red). The panels focus on the dorsal hinge frown region. Scale bar = 5 μm. (TIF) [file pbio.1002536.s008.tif]

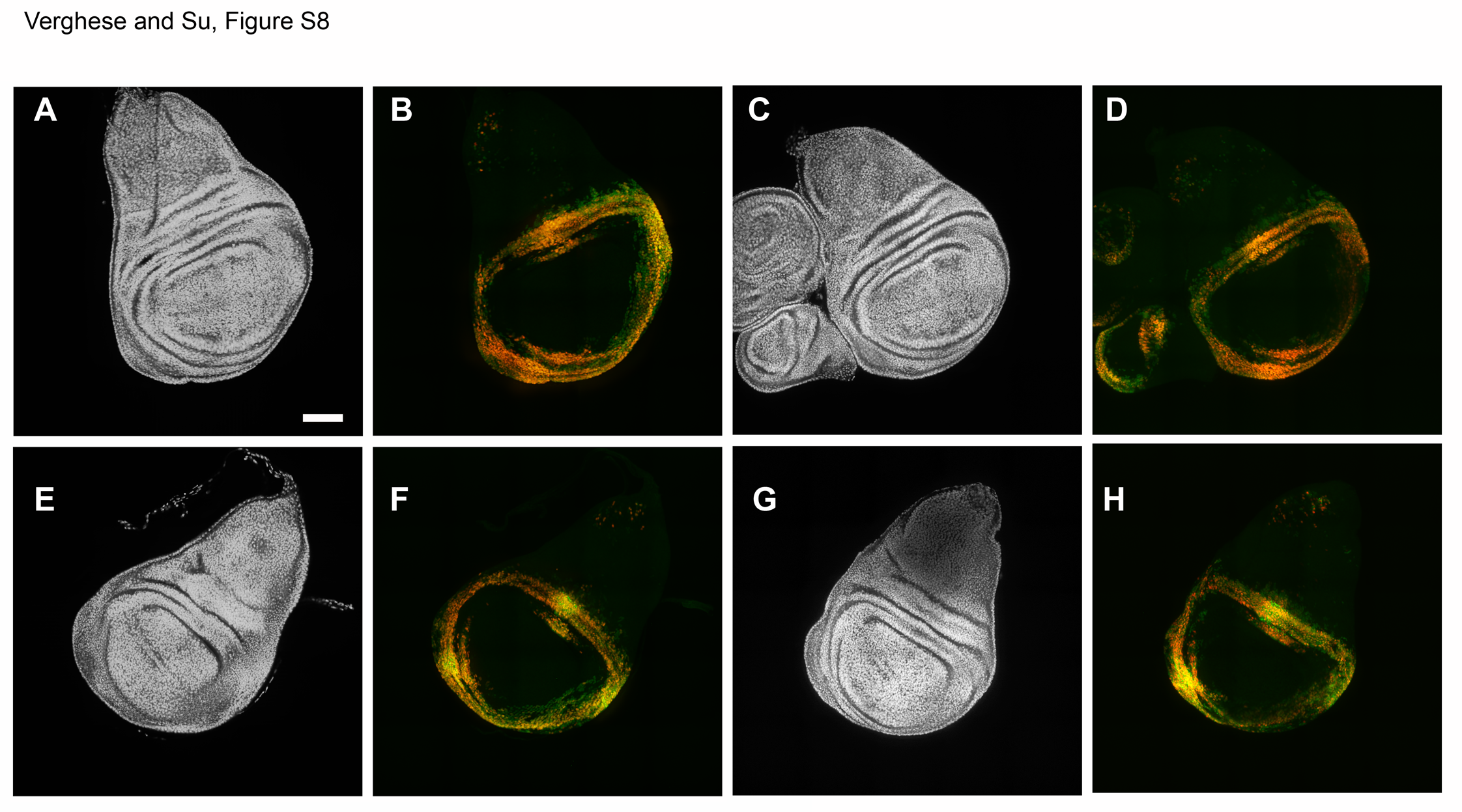

Supplement: S8 Fig — Wing discs were fixed and stained for DNA and imaged for RFP and GFP. The experimental protocol was as in Fig 7A. Larvae were dissected at 24 and 48 h after shift to 29°C, i.e., at the time of irradiation (IR). (A–D) Wing discs from third instar larvae of the genotype UAS-STATRNAi/+; 30A-GAL4>Stringer lineage-tracing chromosome/+; GAL80ts/+. (A, B) 24 h time point; (C, D) 48 h time point. (E–H) Wing discs from third instar larvae of the genotype 30A-GAL4>Stringer lineage-tracing chromosome/+; GAL80ts/UAS-Axin-GFP. (E, F) 24 h time point; (G, H) 48 h time point. Scale bar = 50 μm. (TIF) [file pbio.1002536.s009.tif]

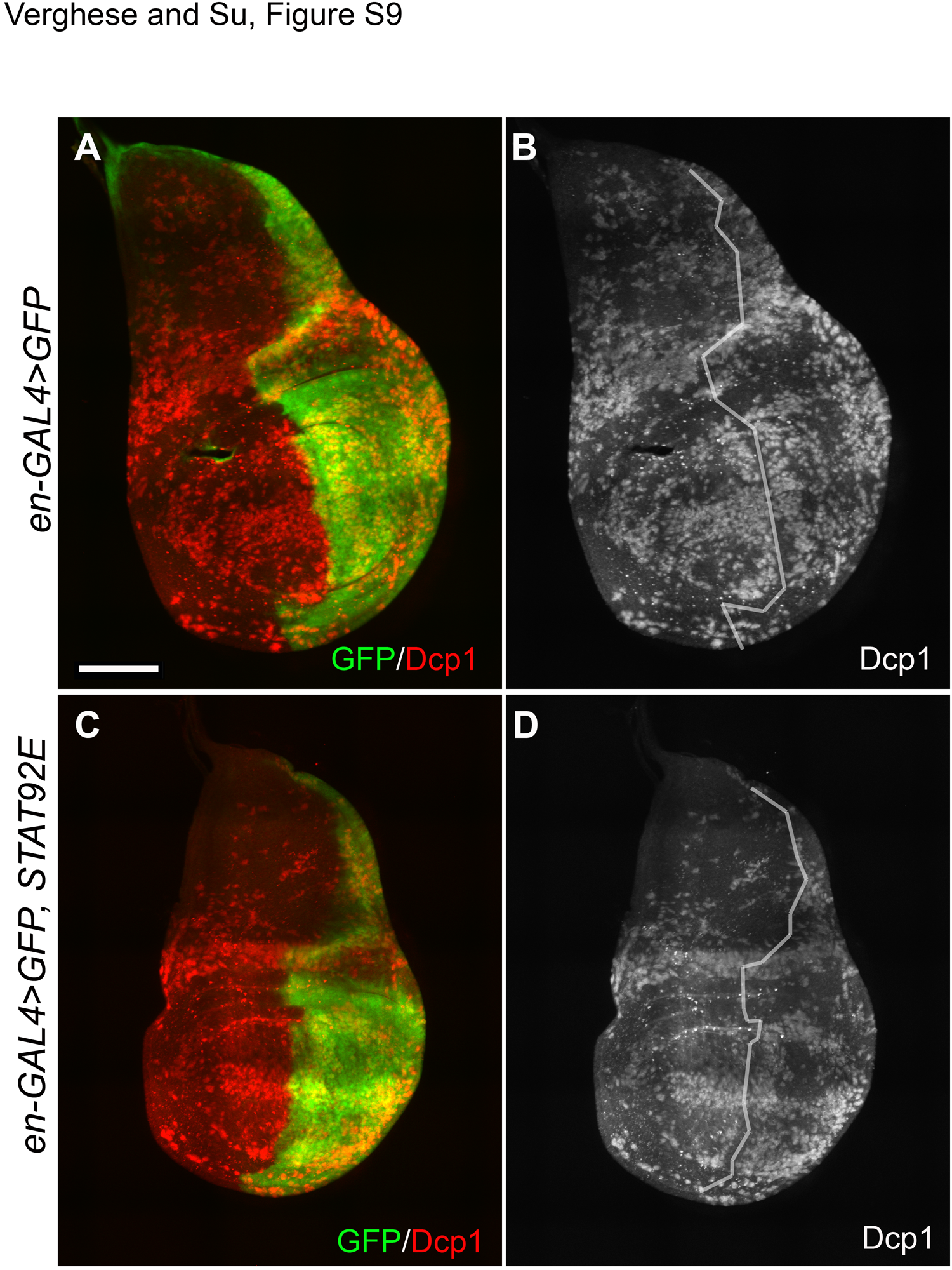

Supplement: S9 Fig — Embryos were collected at 25°C for 8–12 h, reared at 25°C for 96 h from the end of collection, and shifted to 29°C for 24 h to de-repress GAL4 before irradiation with 4,000R of X-rays. Wing discs were dissected 4 h later, fixed and stained for cleaved caspase Dcp1 and DNA, and imaged also for GFP. (A, B) Wing disc from control larvae expressing GFP in the posterior compartment. (C, D) Wing disc from larvae expressing GFP and STAT92E in the posterior compartment. Scale bar = 50 μm. (TIF) [file pbio.1002536.s010.tif]
